# Synthesis of pentameric chlorotin carboxylate clusters for high resolution EUV photoresists under small doses†

**DOI:** 10.1039/d4na00006d

**Published:** 2024-04-09

**Authors:** Cheng-Dun Li, Ting-An Lin, Po-Hsiung Chen, Tsai-Sheng Gau, Burn-Jeng Lin, Po-Wen Chiu, Jui-Hsiung Liu

**Affiliations:** a Department of Chemistry, National Tsing Hua University Hsinchu 30013 Taiwan rsliu@mx.nthu.edu.tw; b TSMC-NTHU Joint Research Center, National Tsing Hua University Hsinchu 30013 Taiwan; c College of Semiconductor Research, National Tsing Hua University Hsinchu 30013 Taiwan

## Abstract

This work reports the synthesis and characterization of a novel pentameric tin chloro cluster, (vinylSn)_3_Sn_2_Cl_5_O_2_(OH)_2_(*t*-BuCO_2_)_6_ (1), and explores its application as an efficient negative-tone photoresist in a 1 : 2 weight ratio blend with [(*n*-BuSn)_12_O_14_(OH)_6_](BF_4_)_2_ (2). Through e-beam lithography, a small high-resolution pattern (HP = 20 nm) is achieved for the blend photoresist (3) at a dose of 2080 μC cm^−2^. Additionally, EUV lithography demonstrates the development of a high-resolution pattern (HP = 16 nm) at an EUV dose of 70 mJ cm^−2^. Mechanistic studies by reflective FTIR indicate a significant decomposition of Sn–carbon and SnO_2_(*t*-Bu) moieties starting at *J* = 35 mJ cm^−2^, which is accompanied by growth of the Sn–O absorption intensity. A collapse of the cluster frameworks of clusters (1) and (2) is observed at *J* > 70 mJ cm^−2^. High-resolution X-ray photoelectron spectroscopy (HRXPS) reveals that low EUV light predominantly decomposes Sn–butyl and Sn–Cl bonds. As EUV doses increase, primary photolytic reactions involve cleavage of Sn–butyl, Sn–O_2_CBu^*t*^, and Sn–vinyl bonds. Notably, the photolytic decomposition of Sn–Cl bonds is distinctive, with only two out of five bonds being cleaved, even at high EUV doses, resulting in a break in film growth at *J* = 27–35 mJ cm^−2^ in the EUV contrast curve. Moreover, HRXPS analysis suggests that radical propagation on the vinyltin end of the blend is unlikely, providing concise mechanistic insights into the photochemical processes governing the behavior of this advanced photoresist.

## Abstract 2

This work reports the synthesis and characterization of a novel pentameric tin chloro cluster, (vinylSn)_3_Sn_2_Cl_5_O_2_(OH)_2_(*t*-BuCO_2_)_6_ (1), and explores its application as an efficient negative-tone photoresist in a 1 : 2 weight ratio blend with [(*n*-BuSn)_12_O_14_(OH)_6_](BF_4_)_2_ (2).

## Graphical abstract

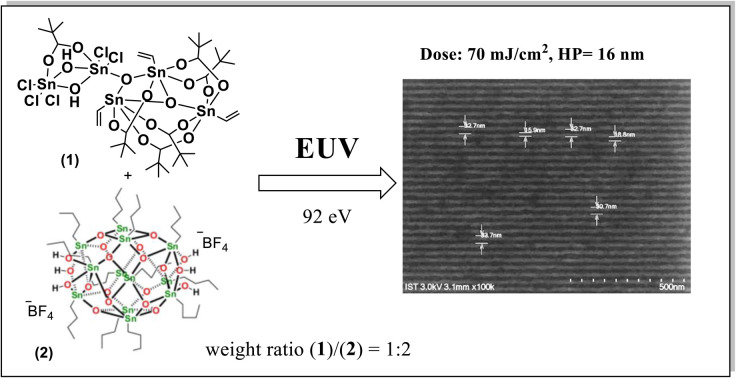

## Introduction

Metal based clusters or nanoparticles are promising extreme ultraviolet (EUV) photoresists to produce a sub-7 nm node integral circuit using EUV lithography.^1–5^ These multinuclear complexes have attracted considerable attention as negative-tone EUV photoresists due to their heightened metal density, enabling more efficient EUV light absorption compared to mononuclear metal complexes. Existing reports on metal clusters or nanoparticles predominantly feature oxide or carboxylate ligands owing to their ready availability.^6–25^ However, oxide ligands exhibit photochemical inertness toward UV light in addition to their weak EUV absorptions. Although carboxylate ligands can be easily decomposed by EUV light, their sizes are somehow too large to facilitate the molecular aggregation of negative-tone photoresists.^14,15^ We have recently prepared a partially decarboxylated hafnium cluster Hf_6_O_4_(OH)_6_(C_4_H_9_CO_2_)_10_ that can achieve a HP = 16 nm pattern under high EUV doses 170–180 mJ cm^−2^.^15^ But another cluster such as Hf_6_(OH)_8_(C_4_H_9_CO_2_)_8_ containing less carboxylated ligands is more photosensitive to achieve a HP = 16 nm pattern with small 30–60 mJ cm^−2^ doses.^14^

While metal clusters incorporating multiple chloro ligands are well-documented,^26^ their application as negative-tone photoresists remains unexplored. Polynuclear chloro clusters present potential advantages, including the susceptibility of weak metal-chloride bonds to cleavage by EUV light, compact molecular packing facilitated by small chloro sizes, and enhanced EUV absorption efficiency compared to oxygen and carbon-based ligands. However, the utilization of metal chloro clusters as EUV photoresists is challenged by the reactivity of M–Cl with surface Si–OH, resulting in the formation of less soluble Si–O–M material.

In this work, we report the synthesis of a novel pentameric tin cluster, (vinylSn)_3_Sn_2_Cl_5_O_2_(OH)_2_(*t*-BuCO_2_)_6_ (1), featuring five Sn–Cl ligands arranged in a highly irregular geometry. While cluster (1) alone is an unsuitable photoresist due to incomplete film removal after a PAB (post-apply bake) treatment, its application in an EUV photoresist is realized through strategic blending. By combining it with another cluster, [(*n*-BuSn)_12_O_14_(OH)_6_](BF_4_)_2_ (2), in a weight ratio of 1 : 2, the resulting blend emerges as an efficient EUV photoresist, enabling the fabrication of a small half-pitch (HP) pattern of 16 nm at a dose *J* = 70 mJ cm^−2^. This performance surpasses that of its major component (2), which necessitates higher energy doses with *J* > 200 mJ cm^−2^ (see Fig. 8, *vide infra*) to achieve a comparable HP = 14–15 nm pattern. Characterization studies, including Fourier-transform infrared spectroscopy (FTIR) and high-resolution X-ray photoelectron spectroscopy (HRXPS), are employed to elucidate the aggregation mechanism of this novel photoresist blend.

## Results and discussion

**Scheme 1:**
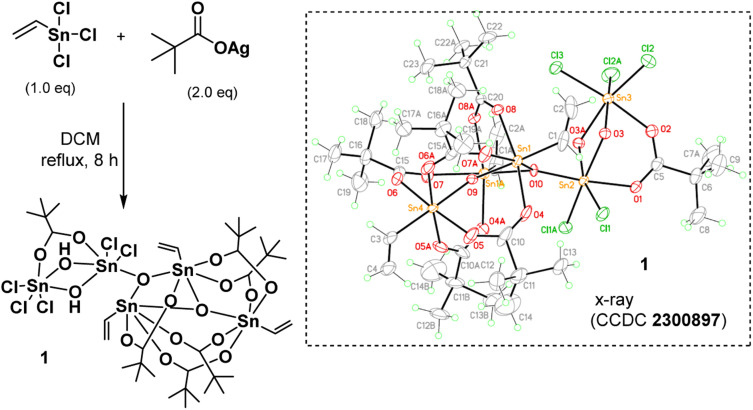
Synthesis and structural characterization of cluster (1).

As shown in Scheme 1, vinyltin trichloride was treated with *t*-BuCO_2_Ag in a 1 : 2 molar ratio (Ag/Sn = 2.0) in hot DCM (40 °C, 8 h), and the resulting solution was evaporated to dryness. Crystallization of this residue in DCM/hexane afforded cluster (1) in crystalline form. The molecular structure of this cluster has been characterized by X-ray diffraction study, revealing a novel structure. The ORTEP image is shown in Scheme 1; notably, the structure consists of two small clusters that are linked by one triply bridged oxide (μ_3_-O). A small cluster contains two tin centers that are coordinated with one carboxylate, two bridging hydroxide and five chloro ligands; the other cluster contains three vinyltin centers that are bound with five carboxylate and two triply bridged oxide ligands. Such a cluster can be formulated as (vinylSn)_3_Sn_2_Cl_5_O_2_(OH)_2_(*t*-BuCO_2_)_6_. This resolved structure is compatible with our elemental analysis, and ^1^H and ^13^C NMR spectra. However, ^119^Sn NMR spectra show one peak at *δ* −32.2 ppm and another peak at *δ* −188.7 ppm at 25 °C in CDCl_3._ As recrystallization of cluster (1) in DCM/hexane (25 °C, 24 h) leads to a 95% recovery; cluster (1) is quite stable in solution. We postulate that cluster (1) has two small tin clusters comprising two and three Sn atoms, respectively. These two subunits shown two ^119^Sn NMR signals as the Sn atoms in each subunit have very close NMR shifts that cannot be resolved.

**Fig. 1:**
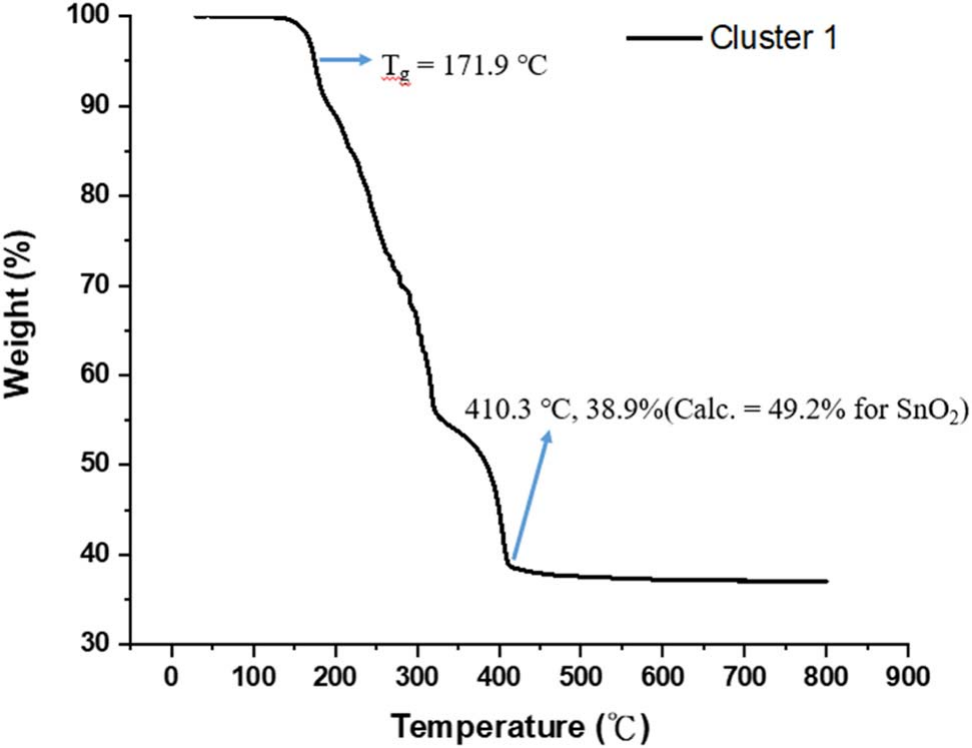
Thermogravimetric analysis in the 30–800 °C range.

Fig. 1 shows the thermogravimetric analysis (TGA) of cluster (1) to study its thermal decomposition process. The cluster is stable below 160 °C, but gradually loses weight from 160 °C to 410 °C at three stages according to the changes in curve slopes. The first stage (160–210 °C) corresponds to a small weight loss (12%), probably due to the loss of water or HCl. The former arises from a dehydration of two Sn–OH groups and the latter occurs with the reaction between the Sn–Cl and Sn–OH bonds. Small decomposition of Sn–carboxylate ligands is also likely to occur here. The second stage (210–312 °C) shows a large weight loss (*ca.* 35%), probably due to the thermal decomposition of Sn–butyl, Sn–vinyl and Sn–carboxylate ligands. A final stage (320–410 °C) is notable, involving a 17 wt% loss, due to unknown reactions. Loss of volatile tin compounds like vinylSnCl_3_, SnCl_4_ or SnCl_2_ likely occurs in this stage because the remaining weight is only 38.9%, far smaller than the expected 49.2% value for SnO_2._ Notably, cluster (1) has the Sn element alone *ca.* 39.6%, showing a large loss of tin species at the end of the TGA curve. The composition of this residue is further studied by XPS study (*vide infra*, see Fig. 11) that shows the following composition for this residue: Sn : C : O : Cl = 5 : 4.9 : 15.2 : 0.5; herein cluster (1) has the following element ratio: Sn : C : O : Cl = 5 : 36 : 15 : 5. Sufficient oxygen content remains in the final residues. Residues with a formula such as SnCl_*x*_O_2–0.5*x*_ are expected to have residual weight >49.2%. With this XPS study, the residue seems to have SnO_2_ as the main component together with a small proportion of Sn–Cl, and Sn–carboxylate residues. However, a loss of volatile tin content is obvious because the remaining weight *ca.* 38.9% is too small.

**Scheme 2:**
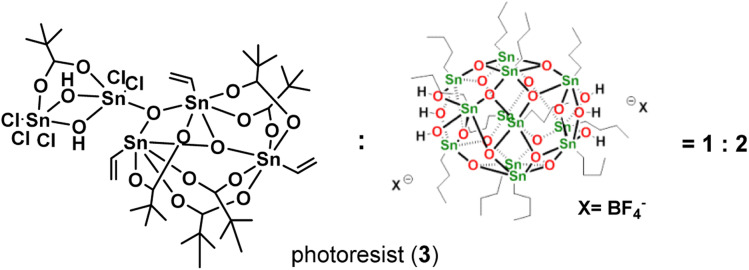
Composition of a new blend photoresist (3).

Our primary focus lies in harnessing the potential of cluster 1 as both an e-beam and EUV photoresist. However, employing this cluster alone results in the formation of an insoluble film, even with a mild PAB process (60 °C, 60 s). The film persists even after treatment with various solvents such as acetone, 2-heptanone, toluene, 4-methyl-1-pentanol, and isopropanol. Although a 1% aqueous solution of tetramethylammonium hydroxide (TMAH) is effective in removing the PAB-treated film, it also removes the e-beam exposure photoresist. To address these challenges, we explore the use of cluster 1 in a blend, combining it with [(*n*-BuSn)_12_O_14_(OH)_6_]X_2_ (X = BF_4_, 2).^27^ The selection of the 12-tin cluster (2) is based on its ability to form a smooth film surface, in contrast to its hydroxide analogue (X = OH), which has been reported to exhibit microcrystalline morphology.^22,28^ Additionally, we are aware that the 12-tin cluster (X = BF_4_, 2) is a very efficient photoresist to develop very high resolution EUV patterns with HP = 12–16 nm under high EUV doses (>200 mJ cm^−2^). Some representative patterns are shown in Fig. 8 (*vide infra*) and S3.† In our investigation of this new blend, we explore weight ratios with (1)/(2) < 0.5, as an increased proportion of cluster (1) in the blend results in incomplete removal of thin films with developers after the PAB treatment (Scheme 2).

**Fig. 2:**
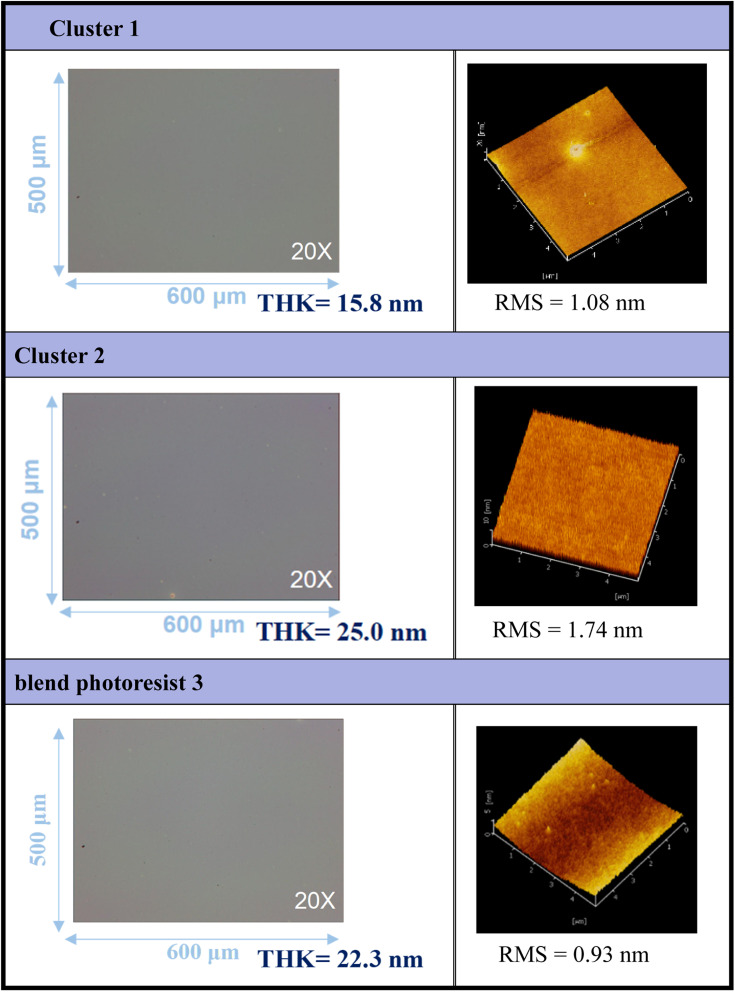
Surface characterization of photoresists (1), (2) and (3) by OPM and AFM; the films are treated by a PAB process (80 °C, 60 s).

Thin films of clusters (1), (2), and their blend photoresist (3) were characterized using optical microscopy (OM) and atomic force microscopy (AFM), and the results are illustrated in Fig. 2. The films were prepared at a concentration of 1.5 wt% in 4-methyl-2-pentanol before spin coating. A PAB process was conducted at 80 °C for 60 s, followed by cooling to room temperature for 24 hours. With the thickness ranging from 15.8 to 25.0 nm, these photoresists exhibited a uniform and featureless appearance, without visible microcrystalline structures observed over the domain of 500 μm × 600 μm, as depicted in the OM images. The surface topography of the films was examined at a higher resolution, revealing remarkably smooth surfaces with roughness values of approximately 1.08 nm, 1.74 nm, and 0.93 nm for photoresists (1), (2), and (3), respectively, over the domain of 5 μm × 5 μm. To assess the solubility of these films, various developers were employed. Notably, photoresist (1) exhibited resistance to complete removal by common solvents, with a residual thickness of 5–6 nm even after two minutes of developer cleaning.

**Fig. 3:**
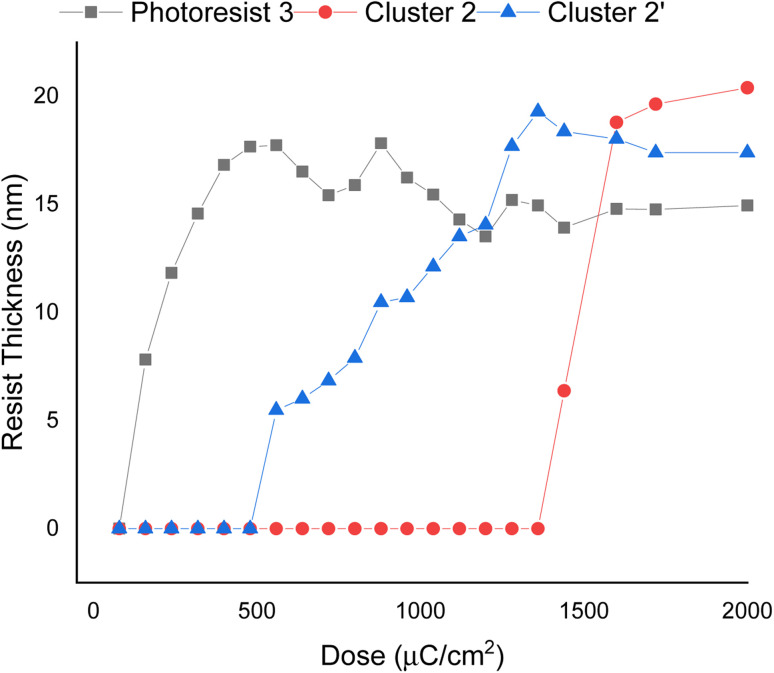
SEM images for e-beam patterns of photoresists 2–3 under PAB (80 °C, 60 s), no PEB, initial thickness: 21 nm, developer: 2-heptanone, 60 s. Cluster 2 refers to the 12-tin cluster (X = BF_4_), and cluster 2′ refers to the cluster with X = OH.

E-beam exposure contrast curves wereobtained to assess the photosensitivity of the thin films of photoresists 1, 2, and 3, following PAB treatment (80 °C, 60 s). No post-exposure bake (PEB) treatment was applied to these photoresists. These curves illustrate the remaining fraction of the exposed resist, not removed by the developer after exposure, as a function of e-beam doses. In Fig. 3, the contrast curve of cluster (1) is unattainable due to a PAB process that reduces solubility of the unexposed photoresist with the developer 2-heptanone. In contrast, the contrast curves of photoresists (2) and (3) exhibit typical patterns for negative-tone photoresists. The critical energy is determined to be 1600 μC cm^−2^ for the 12-tin cluster (2), while this energy is drastically reduced to 500 μC cm^−2^ for the blend photoresist (3). Remarkably, the remaining height after 500 μC cm^−2^ is 17.8 nm for blend (3), representing a modest 15% loss in height compared to its initial height of 21.0 nm. The e-beam photosensitivity of blend material 3 demonstrates that it could be a suitable photoresist due to its low critical energy and a sharp curve slope. We also compared the photosensitivity between 12-tin cluster (2) and the well-known [(*n*-BuSn)_12_O_14_(OH)_6_](OH)_2_ (2′)^25^; the latter has a small critical energy *J* = 1360 μC cm^−2^, but with a small curve slope. In Fig. 3, the height of the exposed photoresist (2′) increases very slowly over a wide range of EUV doses (*J* = 550–1360 μC cm^−2^); this feature is unlikely to produce a high-resolution pattern. Accordingly, we selected 12-tin cluster 2 as the main component to blend with cluster (1).

**Fig. 4:**
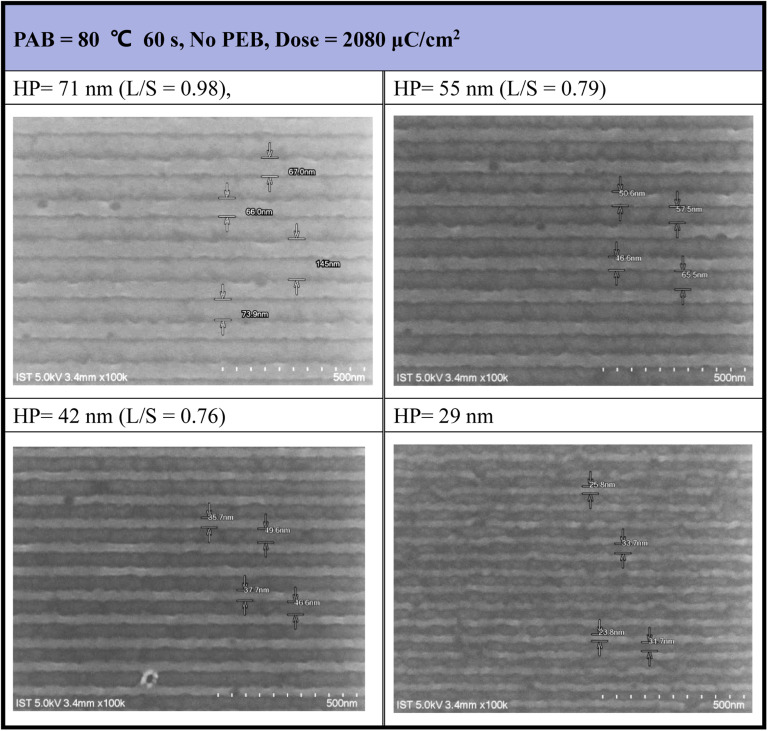
SEM images of e-beam lithography. PAB: 80 °C, 60 s, no PEB, initial thickness 21.0 nm, developer: 2-heptanone (60 s).

**Fig. 5:**
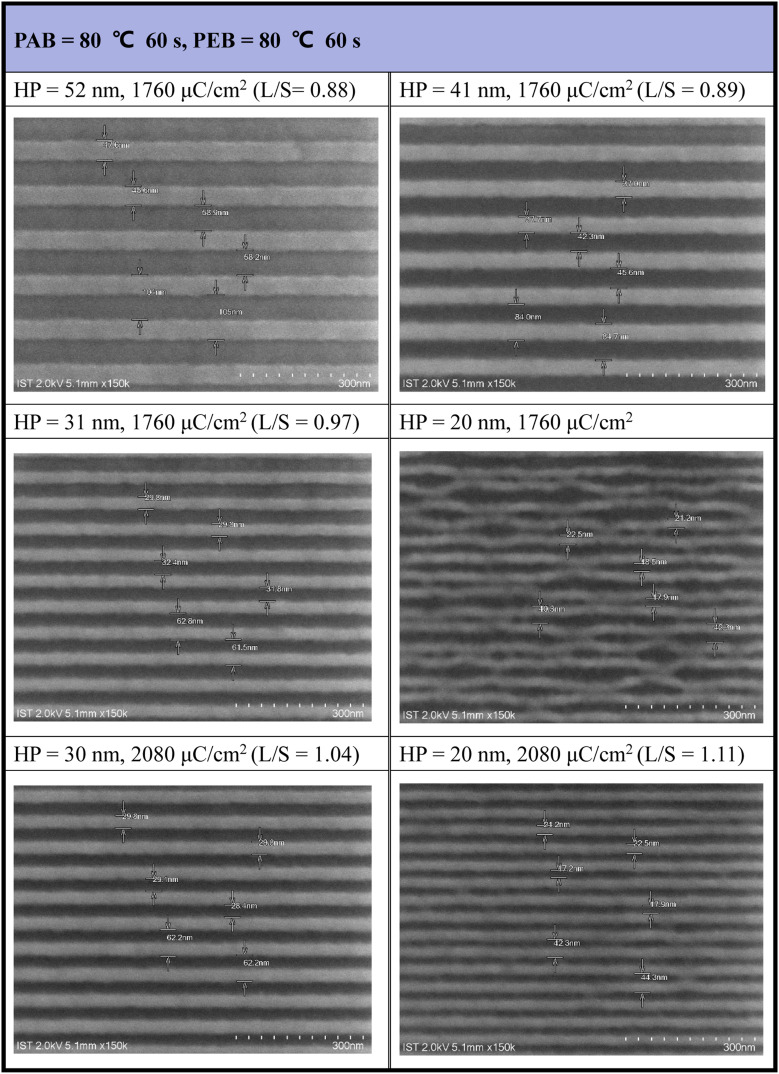
SEM images of e-beam lithographic patterns with PEB (80 °C, 60 s) initial thickness 21 nm, developer: 2-heptanone (60 s).

E-beam lithographic patterns were generated for photoresist (3) under both no PEB and PEB conditions (80 °C, 60 s), with the corresponding images shown in Fig. 4 and 5, respectively. Photoresist 3 underwent e-beam exposure with doses ranging from 450 to 2720 μC cm^−2^ across various resolutions (HP = 70–20 nm). In the absence of PEB, high e-beam doses exceeding 2000 μC cm^−2^ were necessary to resolve a pattern with HP = 30 nm. Fig. 4 illustrates that the line/space ratios decrease with increasing resolutions. Specifically, the *L*/*S* ratio is 0.98 for HP = 71 nm, 0.79 for HP = 55 nm, and 0.76 for HP = 42 nm. This trend indicates inadequate EUV exposure for small half-pitch patterns (HP = 29 nm), resulting in top loss of exposed photoresists. Additional e-beam patterns, featuring various e-beam doses and HP = 20–70 nm patterns, are provided in the ESI (ESI, Fig. S1).†

The efficiency of e-beam lithographic studies was further improved through an additional PEB process at 80 °C (60 s). As shown in Fig. 5, a dose of 1760 μC cm^−2^ can achieve resolution for a HP = 31 nm pattern. In the case of HP = 20 nm, the exposed photoresist 3 exhibits a curved shape in addition to broken lines influenced by the developer (2-heptanone). However, this issue was addressed with higher e-beam doses (*J* = 2080 μC cm^−2^), providing a well-defined pattern for HP = 20 nm. The majority of small patterns (HP = 20–31 nm) exhibited an idealized *L*/*S* = 1, underscoring the high quality of photoresist (3). A gradual increase in the *L*/*S* ratios from a larger HP of 52 nm (*L*/*S* = 0.88) to a smaller HP of 20 nm (*L*/*S* = 1.1) is a typical phenomenon observed in mature photoresists, as photoresist blurring is more noticeable for smaller pitches. Under this PEB condition, additional e-beam patterns, featuring various e-beam doses and HP = 20–70 nm patterns, are presented in Fig. S2 (see the ESI†).

**Fig. 6:**
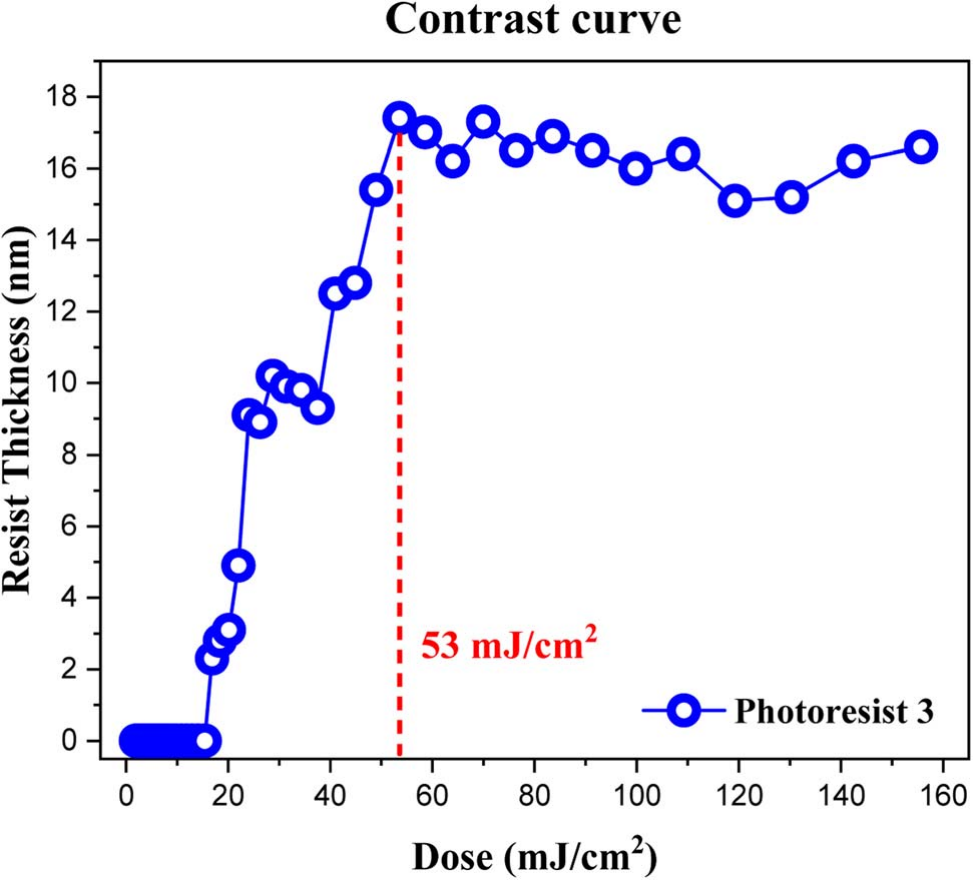
Contrast curve of photoresist (3) using EUV doses. Initial thickness 21.0 nm, developer: 2-heptanone (60 s).

The contrast curve of photoresist (3) was measured under different EUV doses; pattern development and solvent cleaning are the same as those in the e-beam study. EUV-exposure experiments were performed at the Swiss Paul Scherrer Institute Center (PSI) with a standard of error ±1 mJ cm^−2^, and the thickness was measured at α-stepper with an error of ±0.1 nm. PAB was conducted at 80 °C (60 s) before EUV exposure. The curve in Fig. 6 is typical of a negative-tone photoresist. Exposed film thickness starts to increase at *J* = 18 mJ cm^−2^ showing high photosensitivity. Film thickness increases gradually until a maximum height *ca.* 17.8 nm at *J* = 53 mJ cm^−2^. There is a break in film growth in the *J* = 27–36 mJ cm^−2^ interval, showing similar solubility of the film toward the developer. This break seems to be real because there are four EUV doses at this break. Notably, our subsequent HRXPS indicates that high EUV doses can remove carboxylate and vinyl ligands more effectively at *J* > 36 doses, rendering the film less soluble (*vide infra*, Fig. 12 and Table 1). At *J* = 30 mJ cm^−2^, HRXPS indicates a sudden cessation of the Sn–Cl bond cleavage at *J* = 30 mJ cm^−2^ even though three Sn–Cl bonds are still present. This is probably the main reason for a break in film growth in the 27–36 mJ cm^−2^ interval. Similar to its e-beam lithography, a small loss of 15% of thickness is observed relative to its initial height of 21.0 nm.

**Table 1:** The composition of Sn, C, O and Cl elements in the HRXPS of blend (3) at different EUV doses

Dose mJ cm^−2^	Sn	C	O	Cl
Unexposed	19 ± 1	93 ± 1	40 ± 1	5.0 ± 0.1
30	19 ± 1	79 ± 1	42 ± 1	2.8 ± 0.1
60	19 ± 1	75 ± 1	42 ± 1	2.9 ± 0.1
90	19 ± 1	73 ± 1	42 ± 1	2.9 ± 0.1
120	19 ± 1	62 ± 1	43 ± 1	3.0 ± 0.1
150	19 ± 1	58 ± 1	43 ± 1	2.9 ± 0.1

**Fig. 7:**
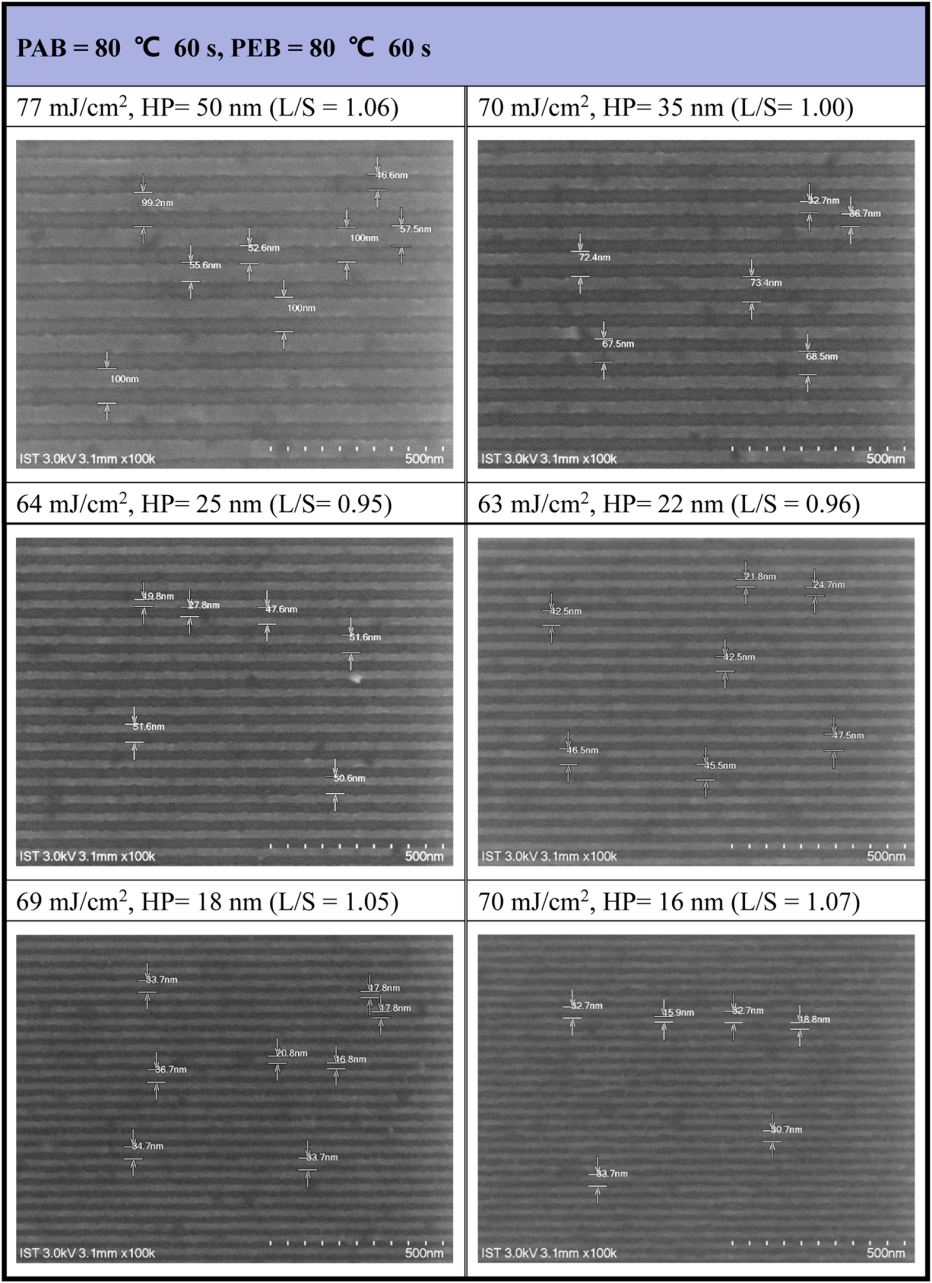
SEM images of EUV patterns for photoresist (3). Initial thickness 22 nm. PAB (80 °C, 60 s) and PEB (80 °C, 60 s), developer 2-heptanone (60 s).

Our next task is to develop the EUV lithographic pattern of photoresist (3) using 2-heptanone as the developer (60 s). An extra PEB treatment (80 °C, 60 s) is applied to save EUV doses. The interference mask at the Swiss PSI includes various HP patterns in the 16–50 nm range. Under small doses (63–77 mJ cm^−2^), as shown in Fig. 7, the SEM images of all developed EUV patterns are well-defined to enable the measurement of line/space (*L*/*S*) parameters. However, few small taints were found on the small HP = 16 or 18 nm pattern, due to inappropriate operation on unexposed photoresist cleaning. This minor problem can be solved with a change to a more soluble solvent such as propylene glycol methyl ether (PGME). The respective *L*/*S* values are as follows: 1.06, 1.00, 0.95, 0.96 and 1.05 and 1.07 for HP = 50, 35, 25, 22, 18, and 16 nm, very close to an idealized ratio (*L*/*S* = 1). As shown in Fig. S3† and 8, the development of a HP = 15 nm pattern for 12-Sn cluster (2) needs EUV doses *J* > 200 mJ cm^−2^, but the energy dose is further reduced to 60–80 mJ cm^−2^ using the blend photoresist (3) comprising a (1)/(2) = 1 : 2 composition. Other EUV SEM images with different doses and half-pitch patterns are provided in the ESI (see Fig. S4).†

**Fig. 8:**
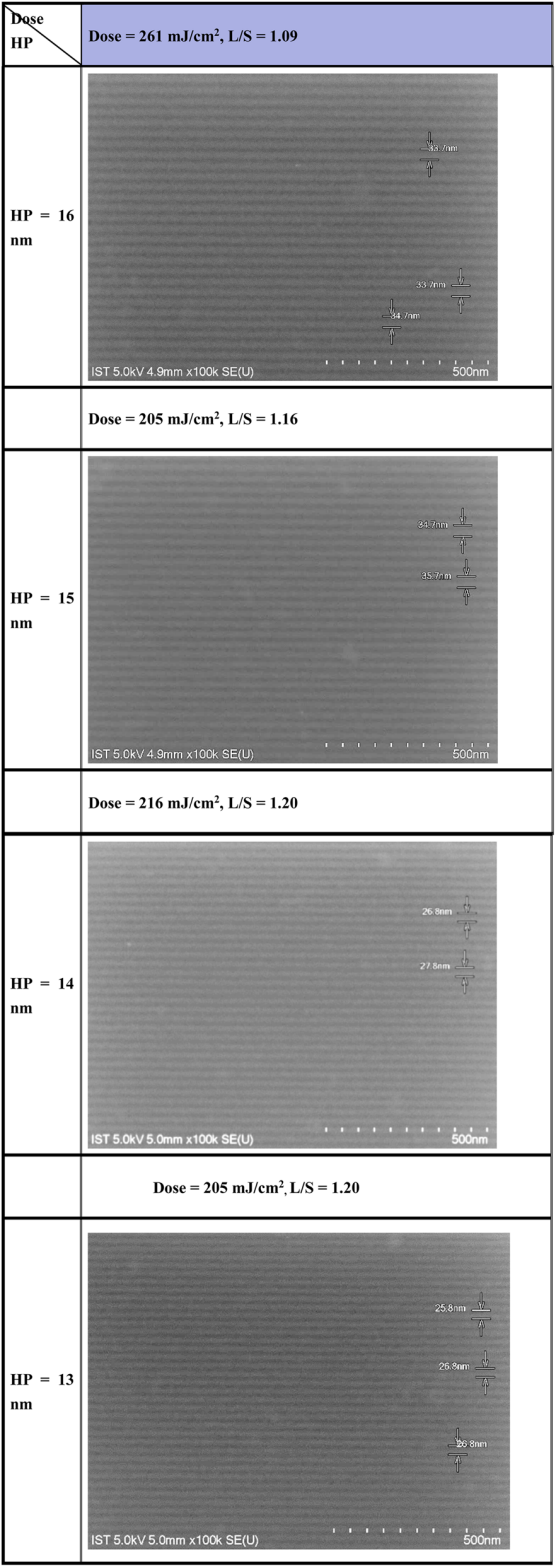
SEM images of EUV patterns for photoresist (2). Initial thickness 22 nm. PAB (80 °C, 60 s) and PEB (160 °C, 60 s), developer 2-heptanone (60 s).

We selected (*n*-BuSn)_12_O_14_(OH)_6_(BF_4_)_2_ (2) as the main component to blend with cluster 1 because this cluster can reach small half-pitch EUV patterns as depicted in Fig. 8 which shows the EUV-pattern of (*n*-BuSn)_12_O_14_(OH)_6_(BF_4_)_2_ (2) which shows superior performance to achieve small half-pitch patterns HP = 13–15 nm, but the corresponding EUV doses are relatively high *J* = 205–216 mJ cm^−2^. In these patterns, there are no broken lines or scums for the exposed photoresists, and the line/space (*L*/*S*) ratios are estimated to be 1.16, 1.20 and 1.20 for the HP = 15, 14 and 13 nm patterns, respectively. These *L*/*S* values are notable because the domains (HP < 15 nm) are very small for common photoresists to form a satisfactory pattern.^29^ Although the EUV patterning of (*n*-BuSn)_12_O_14_(OH)_6_(OH)_2_ (2′) has been previously reported,^25^ the resolution was reported to reach to HP = 30 nm with *J* = 68 mJ cm^−2^.^25^ It is unclear whether this cluster can enable high resolutions with HP < 16 nm.

**Scheme 3:**
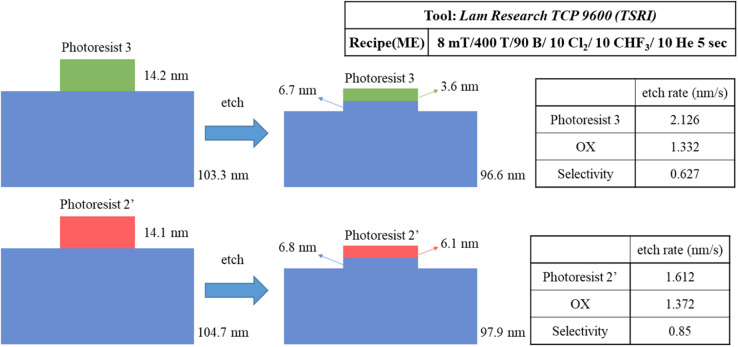
Experimental results for the edge etching of photoresists (2′) and (3).

We prepared two samples for the etching resistance test: the first sample is using our blend sample (3) and the second sample is utilizing photoresist (BuSn)_12_O_14_(OH)_6_(OH)_2_ (2′). Both samples were coated on a ∼100 nm thick SiO_2_ layer and exposed to an e-beam (500 μC cm^−2^ dose) to obtain a 300 × 300 μm^2^ square pattern for subsequent etching tests. With exposure to a mixed gas of Cl_2_/CHF_3_/He for 5 s, the loss of height is used to measure their etching rates; their specific data are shown in Scheme 3. The etching rate of our photoresist (3) is 1.3 times higher than that of (BuSn)_12_O_14_(OH)_6_(OH)_2_ (2′). This leads to the selectivity (OX/PR) exhibiting an acceptable result (sel. = 0.63), which is not significantly different from that of photoresist (2′) (sel. = 0.85). These findings suggest that photoresist (3) shows promising potential in terms of etching resistance. All thickness data were measured using alpha-step and ellipsometer techniques.

**Fig. 9:**
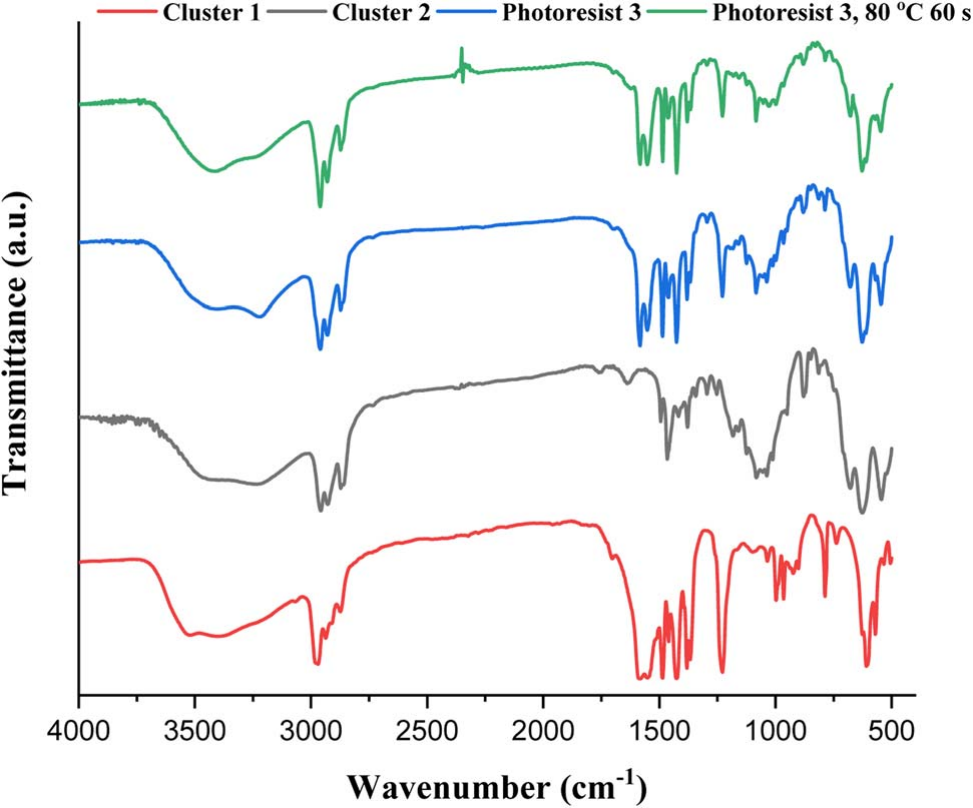
FTIR spectra of photoresists (1)–(3) as KBr pellets, cluster 1 (red) cluster 2 (black), blend 3 (blue) and blend 3 (after 80 °C, 60 s).

IR spectra of photoresists (1)–(3) were studied, and the samples were prepared as KBr pellets. Fig. 9 shows the transmission mode, and there is no new peak for the blend photoresist (3), whose the spectrum is just an overlap of the spectra of two constituents (1) and (2). The absorption peaks for the sp^3^-hybridized *υ*(C–H), *υ*(O_2_CBu^*t*^) and *υ*(Sn–O) stretching modes are labeled for photoresist (3). Notably, the *υ*(C–H) and *υ*(C=C) stretching bands of the Sn–vinyl groups are too weak to observe. The blend material (3) was further baked at 80 °C for 60 s, and its KBr pellet shows an absorption spectrum that is essentially identical to the one without this PAB process. For photoresist (3), the three Sn–O bands at 500–700 cm^−1^ contributed mainly to 12-tin cluster (2).

**Fig. 10:**
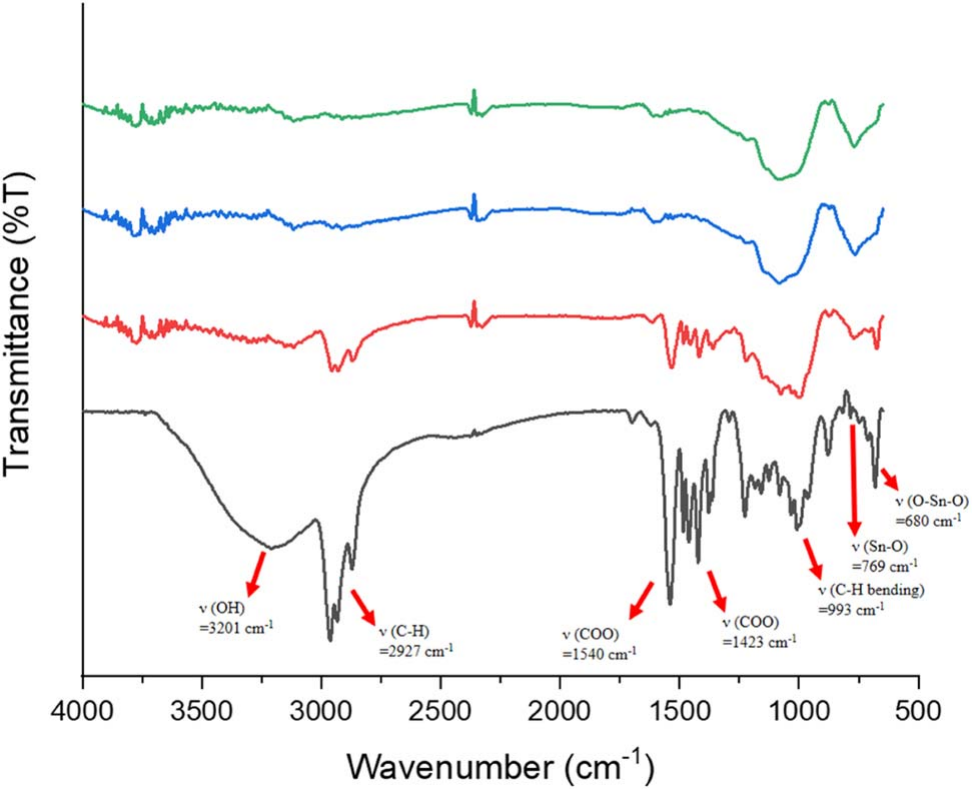
FTIR absorption of photoresist (3) at different EUV doses. From bottom to top: 0, 35, 70 and 100 mJ cm^−2^. The film thickness is measured to be 1.5 μm.

Reflective IR spectra were conducted on the effects of EUV doses on the chemical changes in thin films. A thick film *ca.* 1.5 μm was prepared and the EUV-exposed spot is difficult to locate. Accordingly, the film was briefly rinsed in 2-heptanone for 10 s to locate the exposed spots. We do not exclude the possibility that this exposed spot has been detached from the substrate because the bottom part is still unexposed and removed with 2-heptanone. As shown in Fig. 10, a dose of 35 mJ cm^−2^ led to a sharp decrease in the absorption intensity of the *υ*(OH), *υ*(C–H) and *υ*(O_2_CBu^*t*^) bands centered at 3201, 2920–2980, and 1423 cm^−1^ whereas inorganic bands at 500–900 cm^−1^ exhibit strong IR intensity. This process likely involves a EUV-activated decarboxylation, a dehydration reaction or a Sn–butyl bond cleavage. The relative rates of the carbon loss will be further studied by HRXPS studies. At this *J* = 35 mJ cm^−2^ dose, a small decrease in the *υ*(Sn–O) intensity at 680 nm^−1^ is noted, whereas no new *υ*(Sn–O) band is observed in this region. The framework of 12-tin cluster (2) in this blend material (3) seems to survive. However, at *J* = 70 mJ cm^−2^, most organic residues have been removed by EUV light and only two inorganic bands appear on the film. The band at 1100–900 cm^−1^ is likely due to the absorption of *υ*(Sn–O–Si) whereas a new band at 769 cm^−1^ is assignable to the *υ*(Sn–O–Sn) absorption of SnO_2_-like species.^30–34^ At this high dose, a collapse of the cluster structures of components (1) and (2) is sure to occur because their common band in the 680 nm^−1^ region becomes very weak in absorption intensity; formation of a new broad band at 769 cm^−1^, due to SnO-like species, is observed. These FTIR studies are not able to show the reactivity of Sn–vinyl and Sn–Cl bonds under a EUV dose of *J* = 30 mJ cm^−2^, due to their invisible absorption.

**Fig. 11:**
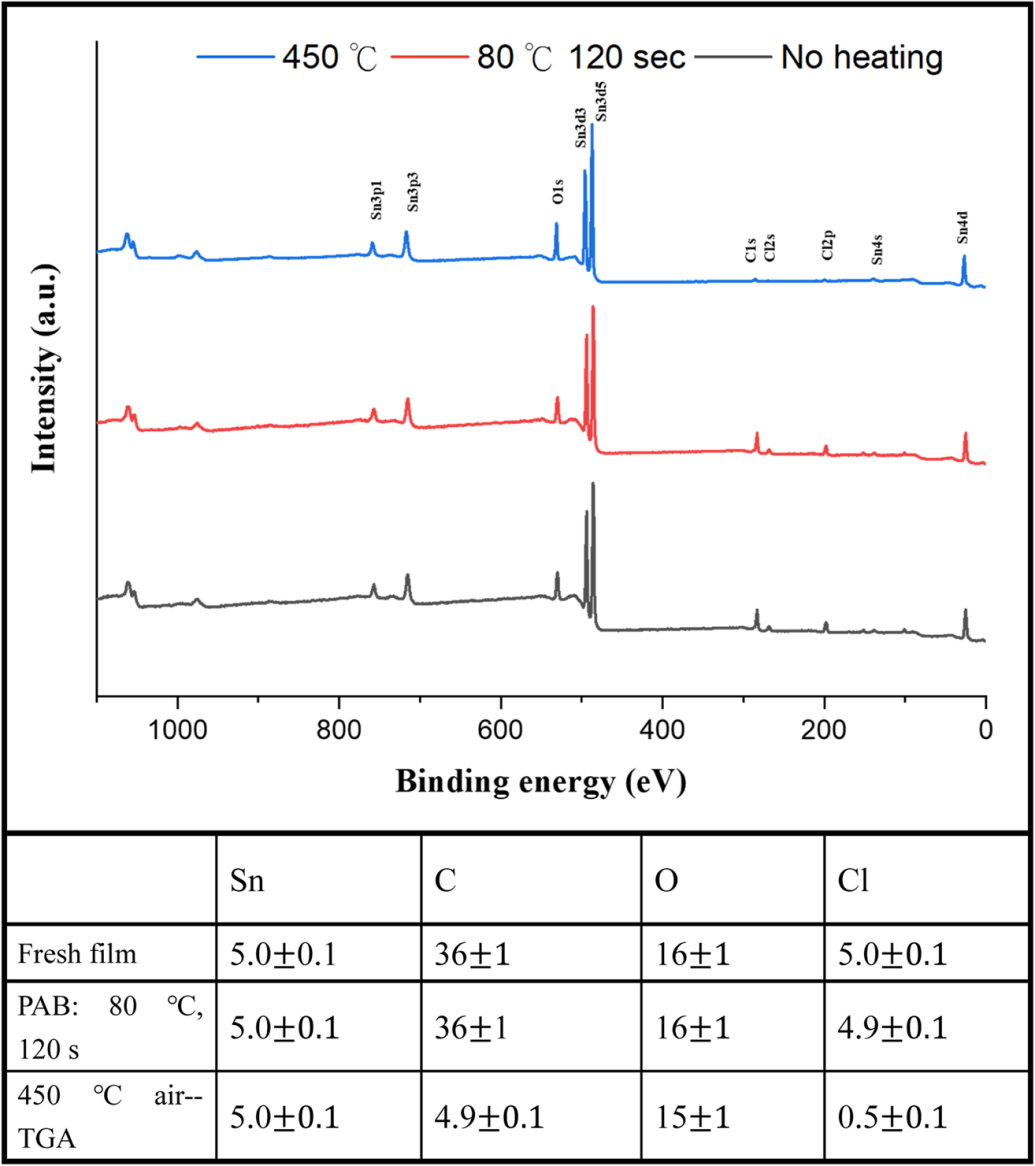
HRXPS of photoresist (1) without PAB (black, bottom), andwith PAB (80 °C, 120 s, red and middle) and the TGA residue after heating at 450 °C (blue line and top).

For photoresist 1, the effect of PAB on surface composition was examined using HRXPS (high resolution X-ray photoelectron spectroscopy) as a tool. A thin film of cluster (1) was prepared in *ca.* 25.0 nm thickness through spin-casting on a silicon wafer; the film was air dried at room temperature for 48 h. Another film was subjected to a PAB treatment (80 °C, 120 s). For these samples, five Sn atoms are used as the internal standard whereas the composition changes of other elements are calculated based on their intensity ratios with respect to the Sn absorption. As shown in Fig. 11, no composition change was observed among Sn, C, O and Cl elements. The Sn–Cl bonds of photoresist 1 seem to be robust toward the PAB process. This assessment is valid only on the surface because these PAB-treatment film still remains at 5–6 nm thickness after cleaning with the developer 2-heptanone (60 s). The Sn, C, O and Cl composition of cluster (1) for the TGA residue was also examined by HRXPS. The sample was treated by the same TGA operation using stepwise heating from 30 °C to 450 °C in air. The residues were pressed into a neat solid film *ca.* 10–12 μm thickness. If Sn content is set at 5 atoms, the loss of carbon and Cl is up to 87% and 90%, respectively. Our TGA study in Fig. 1 also suggests a loss of volatile Sn compounds because the residues are too low in the remaining weight. We also conducted the carbon analysis of the C (1s) carbon component on the TGA residues at 450 °C (see Fig. S5†), and a further band shape fitting indicates that the dominant carbon component is attributed to the sp3-hybridized *t*-butyl group.

**Fig. 12:**
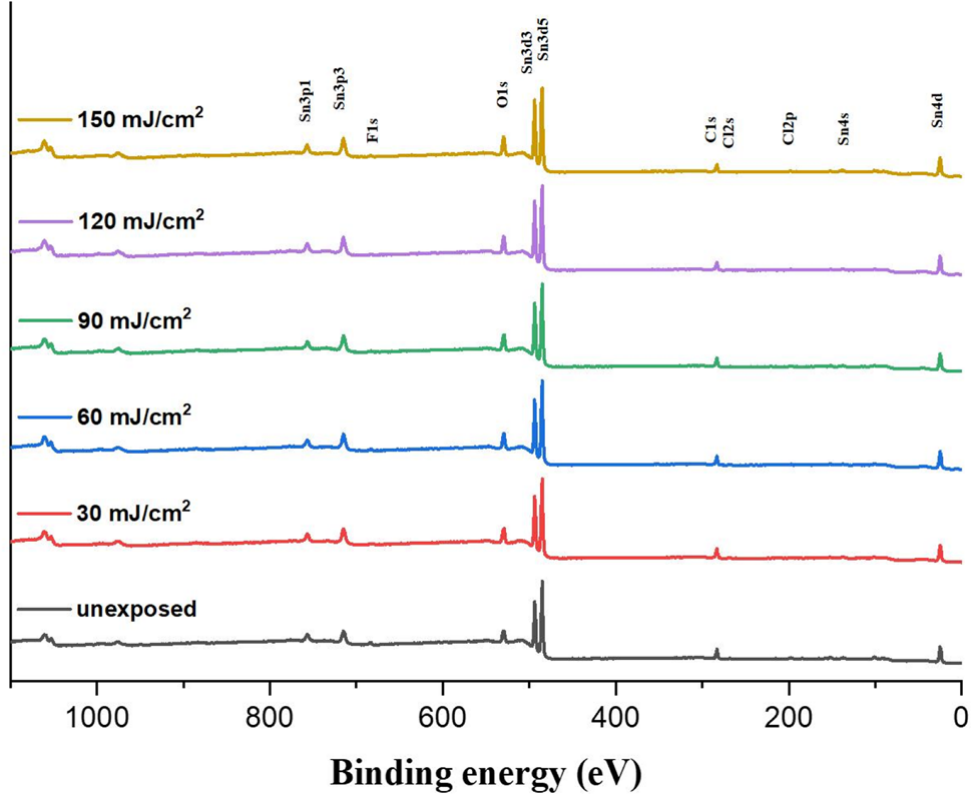
HRXPS of photoresist (3) under different EUV doses (*J* = 0, 30, 60, 90, 120 and 150 mJ cm^−2^).

We investigated the effect of the EUV doses on the element compositions of the film surface using HRXPS as a probe. The film is subjected to a PAB treatment before EUV exposure. This PAB-treated sample is expected to have the same element composition as its fresh thin film because no chemical reactions are shown by IR and XPS study. After EUV exposure at different doses, these samples are exposed to air before storage in a nitrogen-filled container. Fig. 12 shows the HRXPS spectra of the thin films at *J* = 30–200 mJ cm^−2^, which show well-defined absorptions of tin (Sn), carbon (C), oxygen (O) and chlorine (Cl) in the unexposed film. With increasing EUV doses, the absorption intensities of C (1s), O (1s) and Cl (2s) peaks become weak referring to the Sn absorption intensity whereas the boron (B) and fluoride (F) peaks are too weak to see the change. Silicon peaks start to appear at high energy doses (*J* > 90 mJ cm^−1−2^), due to the formation of inorganic Si–O–Sn species; the corresponding IR absorption is also detectable (950–1050 nm^−1^) in Fig. 9. With a weight ratio of (1)/(2) = 1 : 2, the molar ratio of (1)/(2) is 1 : 1.2. With their respective formulae, the element ratios of Sn : C : O : Cl are calculated to be 19 : 93 : 40 : 5.0; herein, chloride atoms are fixed to be five. The initial 19 Sn atoms are used as a standard at different EUV doses. Table 1 summarizes the element compositions at different doses. At *J* = 30 mJ cm^−2^, the carbon content decreased due to the loss of 14 atoms and the chlorine content is lost by 2.2 atoms. In contrast, we observed an increase of the oxygen content with 2 atoms. This process indicates photolytic decompositions of Sn–carbon, Sn–chloride and Sn–carboxylate ligands, forming new Sn–OH or Sn–O–Sn species. With increasing doses from *J* = 40 mJ cm^−2^ to 90 mJ cm^−2^, the carbon loss becomes slow and the oxygen increase is also slow. But there is a rapid loss of carbon atoms with high EUV doses, *J* = 90–150 mJ cm^−2^ whereas the oxygen increase is also relatively large. Our subsequent O (1s) component analysis with HRXPS studies indicate that decomposition of Sn–carboxylate ligands is more rapid at high EUV doses (*vide infra*, Table 3). Notably, the chlorine contents are kept at 2.9–3.0 atoms at *J* = 30–90 mJ cm^−2^, and no further decrease for higher EUV doses; this observation well matches two-stage film growth in the EUV contrast curve (see Fig. 1). There are three different processes corresponding to the loss of carbon atoms, including the EUV-activated decomposition of Sn–butyl, Sn–vinyl and Sn–carboxylate groups. Further analysis of their relative contents relies on HRXPS analysis of the carbon C (1s) component.

**Fig. 13:**
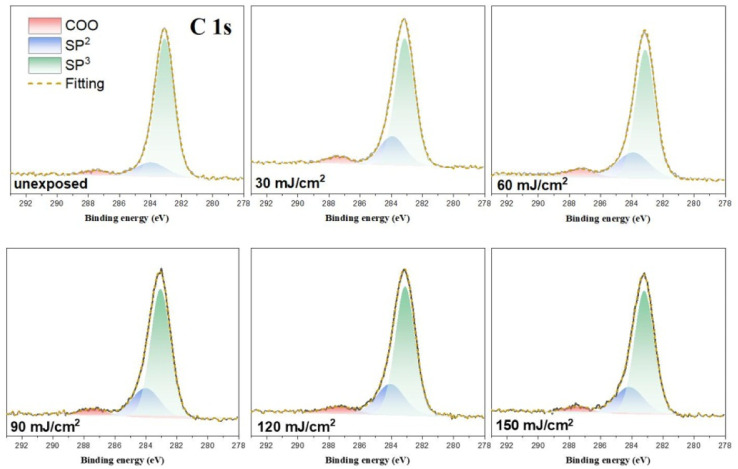
Band shape fitting of the C (1s) components of HRXPS for photoresist (3) under different EUV doses (*J* = 0, 30, 60, 90, 120, 150 cm^−2^).

HRXPS band shape fittings were conducted on the C (1s) and O (1s) absorption peaks to characterize the process of carbon and oxygen change. Band shape fittings are shown in Fig. 13 that also includes their quantitative analysis. Our photoresist 3 contains sp^2^-and sp3-hybridized C–H carbons in addition to the carboxylate C–O carbons. Therefore, only these three carbon components are added together to form the observed C (1s) absorptions at different doses using the software in HRXPS study. In the O (1s) analysis, only two Sn–O and RCO_2_–Sn components are considered as the basis sets. Three components were found for the observed C (1s) peaks, including the sp^3^ and sp^2^ C–C carbons and CO_2_ components centered at 284.0, 284.7 and 287.5 eV, respectively. The sizes of the area in each component are used to calculate their percentages in Table 2. The data in the bracket are the expected values from a blend composition with [1]/[2] = 1.1 : 1.9. The discrepancy is not surprising because the X-ray absorption coefficients are not the same for such different C–H bond modes. Notably, the relative ratios are significantly changed at *J* = 30 mJ cm^−2^, in which the sp^3^-carbon contents are greatly decreased whereas the sp^2^-carbon and carboxylate CO_2_ carbon components have increasing contents. Herein, the main component of sp^3^-carbon decomposition arises from Sn–*n*-Bu, instead of Sn–*t*-Bu. The Sn–*n*-Bu groups are cleaved in greater amounts than those for the SnO_2_C and Sn–CH=CH_2_ carbons. This trend remains unchanged for increasing doses of *J* = 60–90 mJ cm^−2^. This trend is rational because there are 12 *n*-BuSn groups on the tin cluster cage (2) that is known to be very photosensitive due to the high Sn density. On the other hand, radical propagation on the vinyltin moiety is not evident because we do not see a loss of the sp^2^-carbon contents together with an increase in the sp^3^-carbon content. In Table 1, a quick loss of carbon atoms is noted after *J* = 90–150 mJ cm^−2^. In such a high dose stage, the Sn–O_2_C content is increased gradually whereas Sn–CH=CH_2_ content remains approximately the same. Therefore, the SnCH=CH_2_ bonds are cleaved in greater proportion than that for the Sn–O_2_C moieties amid these high EUV doses. Among these carbon components, EUV light decomposes the Sn–O_2_CBu^*t*^ and Sn–vinyl bonds significantly only in high doses whereas the Sn–Cl and Sn–*n*-Bu bond cleavage can readily occur with low EUV doses.

**Table 2:** Band shape fitting of the three C (1s) components of HRXPS of photoresist (3)

Dose (mJ cm^−2^)	COO	CH=CH_2_	*n*-Bu/*t*-Bu
unexposed	4.8 ± 0.1 (6.5 ± 0.1)^a^	14 ± 1 (7.0 ± 0.1)	81 ± 1 (87 ± 1)
30	5.6 ± 0.1	22.±1	72 ± 1
60	5.8 ± 0.1	24 ± 1	71 ± 1
90	5.9 ± 0.1	23 ± 1	71 ± 1
120	6.2 ± 0.1	23 ± 1	70 ± 1
150	6.2 ± 0.1	23 ± 1	71 ± 1

a The number in the bracket is the expected value calculated from a weight ratio of [1]/[2] = 1.1 : 1.9.

**Fig. 14:**
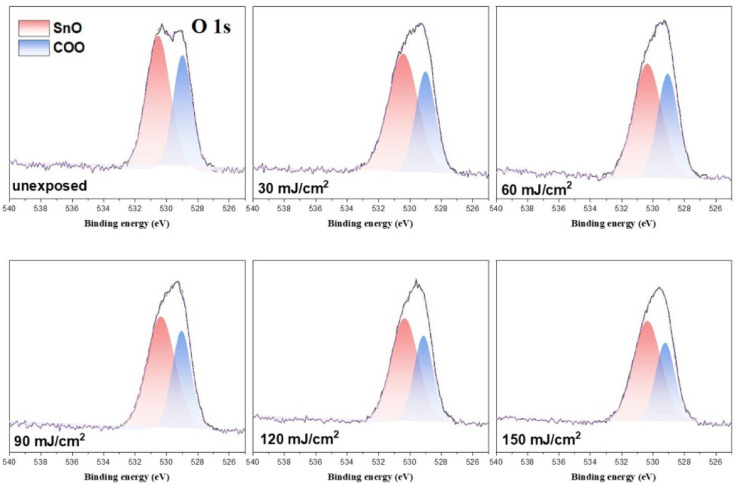
Band shape fitting of the two O (1s) bands of HRXPS of photoresist 3 under different EUV doses. *J* = 0. 30, 60, 90, 120, 150 and 180 mJ cm^−2^.

In the O (1s) peak analysis, the component centered at 530.6 eV in Fig. 14 is assignable to inorganic Sn–O species including Sn_2_OH, Sn_2_O and Sn_3_O;^16,30–32^ the second component centered at 529 eV is assigned to the *t*-BuCO_2_Sn oxygen. Previously, Zhang reported^32^ that the HRXPS spectra of the [(*n*-BuSn)_12_O_14_(OH)_6_]^+2^ core could be resolved into two components such as Sn_3_O and Sn_2_OH in this 530.0–531.2 eV region. But the EUV light *J* > 35 mJ cm^−2^ in our IR study (Fig. 10) shows the collapse of this 12-tin cage (2), possibly forming new species such as Sn_2_O, Sn_4_O and Sn_6_O as in the SnO_2_ solid phase.^33,34^ The use of one single Sn–O component at 530.6 eV is convenient for the band fitting as other Sn–O bands of cluster (1) are also considered. In this study, we prepared the blend (3) containing a weight ratio of (1)/(2) = 1.1 : 1.9, and the ratio of the two components, Sn–O: *t*-BuCO_2,_ is calculated to be 65 : 35; the experimental value is 58 : 42 as shown in Table 3. A PAB process can affect this ratio, but to a small extent (see Fig. 11); this discrepancy is probably due to the different X-ray absorption coefficients of two O (1s) components. As shown in Fig. 14, the increase in the Sn–O content is contributed from the Sn–X bond cleavage (X= Cl and C), further forming the Sn–O bonds of various types in the EUV-exposure/air exposure sequence. However, this oxygen component analysis can detect the degree of carboxylate decompositions at different doses. In the range of *J* = 30–60 mJ cm^−2^, as shown in Table 3, this photolytic process takes place at a slow and steady rate because a large proportion of *t*-BuCO_2_Sn comparison with different dose 30–60 mJ condition have great proportions of O atom than 90–150 mJ. The slow rate of such a *t*-BuCO_2_Sn decomposition is also inferred from the C (1s) analysis in Table 2 and Table 3. At *J* = 30–60 mJ. cm^−2^, the increase in the Sn–O content is also relatively small, *ca.* 61%. But with high doses *J* = 90–150 mJ cm^−2^, the SnO content is increased rapidly, from. 61% to 68% whereas the *t*-BuCO_2_Sn content decreases relatively quickly, from 39% to 33%. Accordingly, high EUV doses are required to decompose the Sn–O_2_CBu^*t*^ bonds.

**Table 3:** Analysis of two O (1s) components from band shape fitting of the HRXPS of photoresist 3

Dose (mJ cm^−2^)	Percentage (%)^a^	
	SnO	CO_2_Sn
unexposed	58 ± 1 (65 ± 1)	42 ± 1 (35 ± 1)
30	61 ± 1	39 ± 1
60	61 ± 1	39 ± 1
90	63 ± 1	36 ± 1
120	66 ± 1	34 ± 1
150	67 ± 1	33 ± 1

a The value in the bracket is the theoretical value using a weight ratio of (1)/(2) = 1.1 : 1.9.

**Scheme 4:**
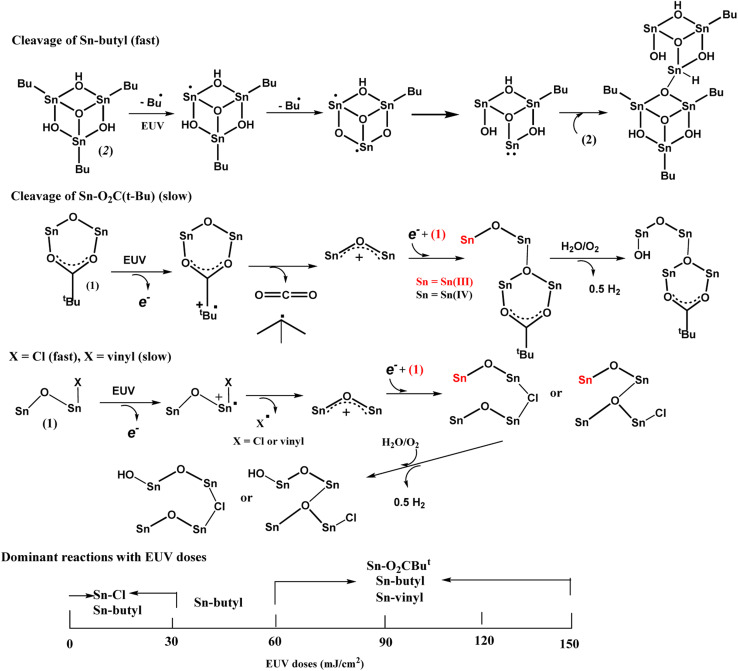
Proposed main reactions with different EUV doses.

The main reactions at different EUV doses are summarized in Scheme 4. With IR and HRXPS studies, we postulate that the photolytic reaction at the low EUV doses at *J* = 30 mJ cm^−2^, involves a quick removal of sp^3^-hybridized carbons (Table 2), mainly from the *n*-butyl groups of 12-Sn clusters. The mechanism of this process has been elucidated by Brouwer and coworkers,^35–37^ involving a direct Sn–butyl bond cleavage, generating Sn(ii)-species for a subsequent aggregation. The Sn–Cl, Sn–vinyl and Sn–O_2_CBu^*t*^ cleavages follow the reported mechanisms of tin carboxylate clusters.^14–16^ Although the photolytic decomposition of Sn–O_2_CBu^*t*^ and vinyltin groups also takes place for the blend (3), their rates are slow because a large proportion still remains at *J* = 60 mJ cm^−2^ as shown in Table 3. For the vinyltin groups, radical propagation at this functionality is not supportive. A summary of the three functionalities in EUV photochemistry is provided below. We postulate that at low doses of 30–60 mJ cm^−2^, HRXPS analysis on the C (1s) component in Table 2 indicates a quick cleavage of Sn–butyl and Sn–Cl bonds. At *J* = 60 mJ cm^−2^, 2–3 Sn–butyl bonds and two Sn–Cl bonds have been cleaved by EUV light. Photolytic decomposition of Sn–O_2_CBu^*t*^ and vinyltin groups becomes significant only at high EUV doses (*J* = 90–150 mJ cm^−2^).

Photolytic decarboxylation of Sn(O_2_CBu^*t*^) and Sn–vinyl moieties is relatively slow as compared to the Sn–Cl and Sn–butyl cleavage according to the C (1s) analysis in Table 2. A fast cleavage of Sn–butyl and Sn–Cl bonds is rational because the sp^3^-hydridized Sn–C and Sn–Cl bonds are generally weak. No further Sn–Cl bond cleavage is noted at high doses *J* = 60–150 mJ cm^−2^. One possible reason is to form a Sn–Cl–Sn bridge that will be very difficult to dissociate because of two coordinated Sn centers. Unfortunately, a band shape fitting of Cl (2s) or Cl (2p) is not satisfactory because the peaks are very small.

Our HRXPS studies can explain the two-stage rise in the EUV contrast curve in Fig. 6. A break in the *J* = 27–36 mJ cm^−2^ region is associated with a sudden cessation of the Sn–Cl bond cleavage so that only Sn–butyl bond cleavage is occurring at these doses. The main reactions in different E|UV doses are shown in Scheme 3.

FTIR spectra also provide mechanistic insight; one novel finding is the collapse of the cluster structures of 12-tin cage (2), and 5-tin-5-Cl carboxylate species (1) is also observed starting at *J* = 70 mJ cm^−2^, forming SnO_2_-like species. Furthermore, increasing EUV doses significantly decreases the IR absorption intensity that is accompanied by increasing intensity of Sn–O bonds. Only a small proportion of organic moieties remains at a high dose at *J* = 150 mJ cm^−2^.

## Conclusions

Prior to this work, development of negative-tone EUV photoresists was exclusively based on metal carboxylate or metal oxide clusters. Metal clusters containing multi-chloro ligands are also very abundant among metal complexes. Since metal–chloride bonds are weak, such complexes in cluster forms are potentially useful photoresists. This work reports the synthesis of a novel pentameric tin chloro cluster (1) that has been well characterized by appropriate physical methods. After a mild PAB process, the film of the cluster (1) cannot be cleaned completely by various solvents. Its mixture with [(*n*-BuSn)_12_O_14_(OH)_6_](BF_4_)_2_ (2) in a 1 : 2 weight ratio proves to be an efficient negative tone photoresist. In an e-beam lithographic pattern, the blend can produce a small HP = 20 nm pattern with a 2080 μC cm^−2^ dose; its EUV lithographic pattern can be developed with a high resolution pattern (HP = 16 nm) under a low EUV dose, *J* = 70 mJ cm^−2^. Reflective FTIR reveals a quick decomposition of Sn–C and Sn–O_2_C(*i*-Bu) starting at *J* = 35 mJ cm^−2^. With EUV doses >*J* = 70 mJ cm^−2^, a collapse of the cluster frameworks of clusters (1) and (2) is clearly observed because their common Sn–O absorption bands at around 680 nm^−1^ become very weak in absorption intensity. With HRXPS study, our mechanistic investigation reveals that EUV light decomposes the Sn–butyl and Sn–Cl bonds predominantly at low EUV doses. With increasing EUV doses, the main photolytic reactions involve the Sn–butyl, Sn–O_2_CBu^*t*^ and Sn–vinyl bond cleavages. Photolytic decomposition of Sn–Cl bonds is noteworthy because only two Sn–Cl bonds are cleaved and the remaining three Sn–Cl bonds are robust even at high EUV doses. This mechanism process might explain a break of film growth at *J* = 27–35 mJ cm^−2^ in the EUV contrast curve of blend 3.

## Experimental section

### Material preparation and characterization

All chemicals were obtained from Sigma company except tetravinyltin which was bought from TCI company. FTIR spectroscopic studies of powder samples were performed using a Bruker Vertex 80v spectrometer. ^1^H, ^13^C NMR and ^119^Sn spectra were recorded on a Bruker 400 or 500 MHz spectrometer using chloroform d–d_1_ (CDCl_3_) as the internal standard. The chemical shifts of ^119^Sn NMR spectra are reported with Me_4_Sn as the reference (*δ* 0.0 ppm). TGA was measured on a Mettler-Toledo 2-HT at a heating rate of 8 °C min^−1^.

### Thin-film deposition

Clusters 1 or 2 and photoresist 3, at 1.5 wt% were dissolved in 4-methylpentan-2-ol; the solution was filtered through a 0.22 μm PTFE syringe filter. The film was deposited by spin-coating at 1200 rpm for 10 s and 1600 rpm for 25 s on a silicon wafer coated with a SiO layer (THK = 100 nm). The wafer was baked at 80 °C for 60 s, respectively, for all three samples. The thickness of the thin films was in the range of 15.8–25.0 nm, which was measured by using a J. A. Woollam M2000. Atomic force microscopy (AFM) images were obtained with a SEIKO SPA-300 HV, using the contact mode. These films were also used for e-beam and EUV exposure.

### Electron-beam lithography (EBL)

Electron-beam lithography was carried out on an Elionix ELS-7800 with an accelerating voltage of 80 kV, and a beam current of 200 pA for the contrast curve and 50 pA for the line pattern. After e-beam exposure, the samples were developed with 2-heptanone for 60 s and rinsed with deionized water. To obtain the contrast curve of the photoresist, a series of squares (50 × 50 mm^2^) were prepared, each with e-beam dosages varied from 80 μC cm^−2^ to 2720 or 2400 μC cm^−2^. The contrast curve was then obtained by measuring the remaining thickness of each exposed square area through an α-step tool after solvent development. To analyze the resolution limit of our resists, different dense line features are designed from 50 nm HP down to 20 nm HP, and the dosages to obtain the best resolution pattern were optimized at 2080 and 1760 μC cm^−2^, respectively, for photoresist 3.

### EUV-IL exposure

Periodic aerial images were generated by two-beam interference in an EUV interference lithography (EUV-IL) system. The EUV-IL exposure at the Swiss Light Sources (SLS), Paul Scherrer Institute, involves 13.5 nm light with a high spatial coherence length and uniform illumination, to transmit a grating mask. This mask comprises multiple grating pairs with periods ranging from 100 nm HP to 32 nm HP, and the period of the first-order interference on the resist is half that on the mask grating. Dosage on the mask (Dosemask) ranges from 40 mJ cm^−2^ to 1211 mJ cm^−2^ with 50 mJ cm^−2^ increments, and the dosage on the resist (Doseresist) needs to divide Dosemask with tool factors corresponding to various grating pairs. The samples for XPS and ATR-FTIR study were exposed to EUV light without any mask at the National Synchrotron Radiation Research Center, Taiwan (NSRRC), TLS 21B2.

### Pattern development

The films after e-beam or EUV exposure were baked at 80 °C for 60 s before cooling at room temperature. The pattern was developed with 2-heptanone for 60 s before baking at 90 °C for 90 s.

### Measurement of edge etching rates

A Lam Research TCP 9600 was employed as a chamber to measure the etching rates. The samples of photoresists (3) and (2′) are prepared by e-beam and mounted on the SiO_2_ layer, as described in the text. The samples were evacuated to reach 8.0 × 10^−3^ torr under the condition of 400 W/90 bias. A stream gas of Cl_2_/CHF_3_/He, each at a 10 sccm flowing rate, was passed through the samples for 5 s. All thickness data were measured using alpha-step and ellipsometer techniques.

### FT-IR measurement

A thin film of a blend photoresist 3 was coated on a 2.4 × 5 cm^2^ silicon wafer by spreading a 4-methylpentan-2-ol solution (3.5 wt%, 0.9 mL) over this wafer substrate; the thickness is measured to be 1.5 nm. This wafer was dried in air at room temperature for 48 h. The coated film was then baked at 80 °C for 60 s. After EUV exposure, this film was developed with 2-heptanone for 10 s to locate the exposed area before further baking at 90 °C for 90 s before FT-IR measurement. The operation was performed in air on a Bruker model Tensor 27 equipped with a KBr beam splitter. The signals were collected in transmission mode with an MCT (mercury cadmium telluride) detector; the resolution is 4 cm^−1^.

### High resolution X-ray photoelectron spectroscopy (HRXPS)

HRXPS data were measured on a ULVAC-PHI Quantera II, with a monochromatic Al K_α_ source (energy of 1486.7 eV). A survey spectrum was obtained with a pass energy of 280 eV and an energy step of 1 eV; a pass energy of 55 eV and energy step of 0.1 eV were used for O, C, and Sn high-resolution spectra. Thin films were prepared with 25–30 nm thickness and baked at 80 °C for 60 s for EUV exposure at the NSRRC center, Taiwan. After exposure, standard development was performed with 2-heptanone for 60 s before baking at 90 °C for 90 s. This film is used for HRXPS study.

### Procedure for synthesis of cluster (1)

Vinyltin trichloride (0.783 g, 3.11 mmol) and silver pivalate (1.30 g, 6.22 mmol) were heated and refluxed in dichloromethane (20 mL) for 8 hours. The solution was filtered through a celite bed, and concentrated and recrystallized in a dichloromethane/*n*-hexane mixed solvent, ultimately affording colorless crystals of cluster 1 (245 mg, 0.138 mmol, 22.3% yield). X-ray crystallographic data, ^1^H, ^13^C and ^119^Sn NMR spectra and their spectral data are provided in the ESI.†

### Procedure for synthesis of (*n*-BuSn)_12_O_14_(OH)_6_(BF_4_)_2_ (2)

To a THF solution (11.2 mL) of (*n*-BuSn)_12_O_14_(OH)_6_(OH)_2_ (1.12 g, 0.454 mmol)^22,25^ a THF solution (0.75 mL) of HBF_4_ (169 mg, 0.96 mmol) was added, and the resulting solution was stirred at 25 °C for 1.0 h. The solution was concentrated and evaporated to dryness *in vacuo*. The resulting white solid was washed with diethyl ether to obtain the pure form of cluster 2 (1.04 g, 0.397 mmol, 87.5% yield). ^1^H, ^13^C and ^119^Sn NMR spectra and their spectral data are provided in the ESI.†

## Author contributions

J.-H. Liu was responsible for the design of all synthetic work. T.-S. Gau and B.-J. Lin were in charge of the lithographic development. P.-W. Chiu and B.-H. Chen conducted e-beam lithographic work. C.-D. Li and T.-A. Lin performed the synthesis and characterization of the three photoresists. Jui-Hsiung Liu and Rai-Shung Liu refer to the same person. The former is the passport name of Rai-Shung Liu; the patent laws of Taiwan and USA only allow passport names for patent applications.

## Conflicts of interest

The authors declare no conflict of interest.

## Supplementary Material

NA-006-D4NA00006D-s001

NA-006-D4NA00006D-s002
