# Supplementary material for: Synthesis of pentameric chlorotin carboxylate clusters for high resolution EUV photoresists under small doses
Source: Nanoscale Adv. 2024 Apr 9;6(11):2928–44. doi: 10.1039/d4na00006d (PMC11134253; doi:10.1039/d4na00006d)
Supplement: NA-006-D4NA00006D-s001 [file NA-006-D4NA00006D-s001.pdf]

Electronic Supplementary Material (ESI) for Nanoscale Advances.
This journal is © The Royal Society of Chemistry 2024

Supporting Information

Synthesis of Pentameric Chlorotin Carboxylate Clusters for High Resolution EUV Photoresists Under Small Doses

Cheng-Dun Li,[a] Ting-An Lin,[a] Po-Hsiung Chen,[b] Tsi-Sheng Gau[b,c] Burn-Jeng Lin[b,c] and Jui-Hsiung Liu*[a]

Keywords: Pentameric chlorotin carboxylate clusters, negative-tone photoresists, high resolution patterns, e-beam and EUV lithograpgy, FTIR and HRXPS studies

Department of Chemistry,[a] TSMC-NTHU Joint Research Center,[b] Colleage of Semiconductor Research[c], National Tsing Hua University, Hsinchu 30013, Taiwan
e-mail:rsliu@mx.nthu.edu.tw

Content:

1. Representative Synthetic procedures -- 1
2. SEM images for e-beam lithography patterns -- 2
3. SEM images for EUV lithography patterns --5
4. Spectral data of key compounds ---11
5. X-ray crystallographic data of cluster 1 --11
6. ^{1}H, ^{13}C and ^{119}Sn NMR of cluster 1 ---26
7. ^{1}H, ^{13}C, ^{19}F and ^{119}Sn NMR of cluster 2 ---29

1. Representative synthetic procedures

Unless otherwise noted, all reactions were carried out under nitrogen atmosphere inoven-dried glassware using standard syringe, cannula and septa apparatus. Dichloromethane and toluene were dried over CaH2 and distilled. Reagents were purchased from commercial sources and used without purification, unless otherwise stated. 1 H NMR and 13C NMR spectra were recorded on a Bruker 400 MHz and Bruker 500 MHz spectrometers using chloroform-d (CDCl3) as the internal standard. The ESI-Mass were perdormed using JEOL JMS-700. The EA analysis was performed by elementar vario EL cube. The TGA were performed using Mettler-Toledo 2-HT. FTIR Spectroscopy of power samples was in a Bruker Vertex 80v spectrometer. The AFM measurements were using SEIKO SPA-300HV. Electron-beam lithography was done by utilizing Elionix ELS-7800 with an accelerating voltage of 80 kV and a beam current of 200 pA. The EUV-IL system at the Swiss Light Sources (SLS), Paul Scherrer Institute, utilizes 13.5 nm EUV light. HRXPS measurements were performed in a ULVAC-PHI Quantera II, with a monochromatic Al Kα source (energy of 1486.7 eV).

2.SEM image of E-beam lithography patterns

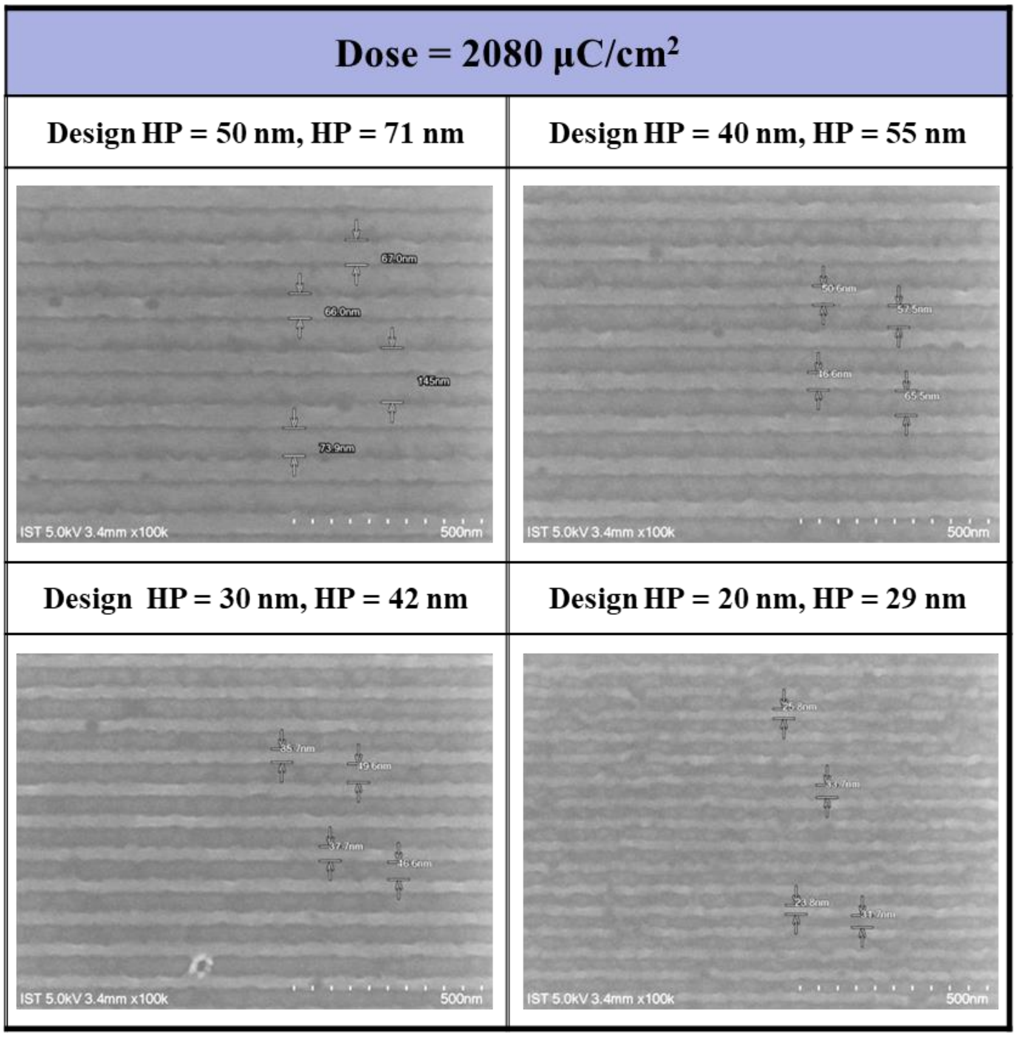

HP \ Dose	Dose = 2440 μC/cm², HP = 64 nm	Dose = 2720 μC/cm², HP = 69 nm
**Design HP = 50 nm**	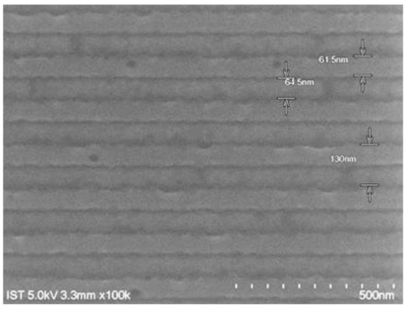	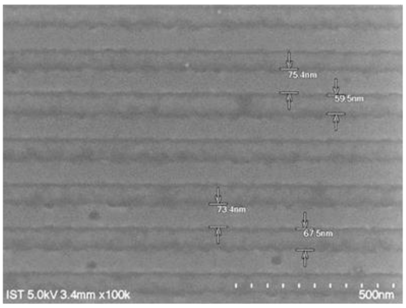
	**Dose = 2440 μC/cm², HP = 56 nm**	**Dose = 2720 μC/cm², HP = 54 nm**
**Design HP = 40 nm**	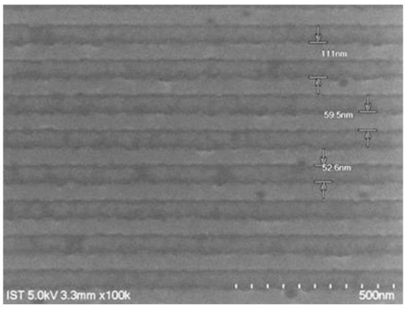	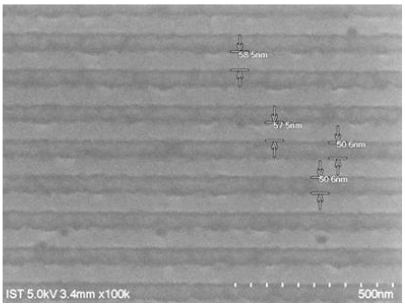
	**Dose = 2440 μC/cm², HP = 40 nm**	**Dose = 2720 μC/cm², HP = 39 nm**
**Design HP = 30 nm**	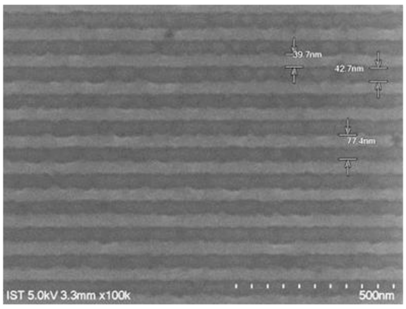	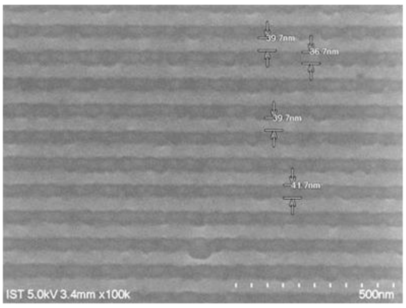
	**Dose = 2440 μC/cm², HP = 27 nm**	**Dose = 2720 μC/cm², HP = 27 nm**
**Design HP = 20 nm**	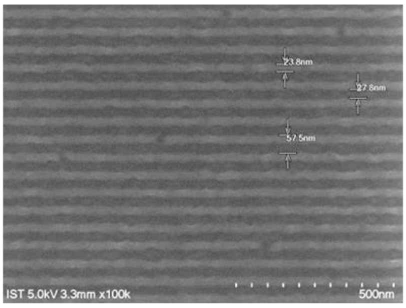	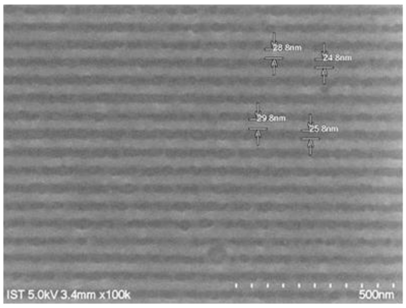

Figure S1. SEM images of E-beam lithography patterns on photoresist 3. Process parameter: 1.5 wt%, thickness 22.3 nm, Developer: 2-Heptanone 60 s, no PEB. (Dose = 2080, 2440 and 2720 μC/cm²).

HP \ Dose	Dose = 800 $\mu C/cm^2$	Dose = 1120 $\mu C/cm^2$,<br>HP = 54 nm
**Design HP = 50 nm**	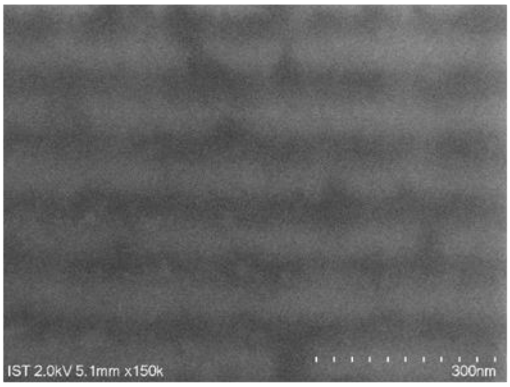	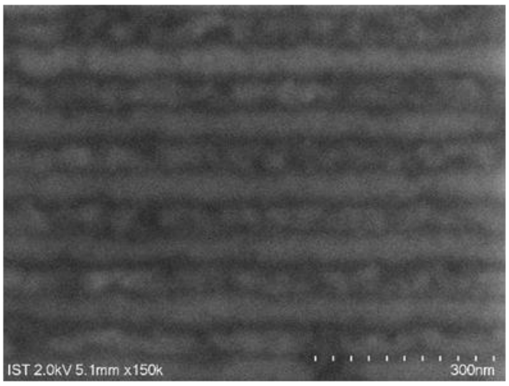
	**Dose = 800 $\mu C/cm^2$**	**Dose = 1120 $\mu C/cm^2$**
**Design HP = 40 nm**	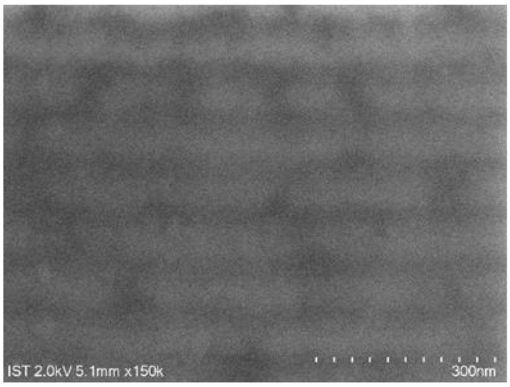	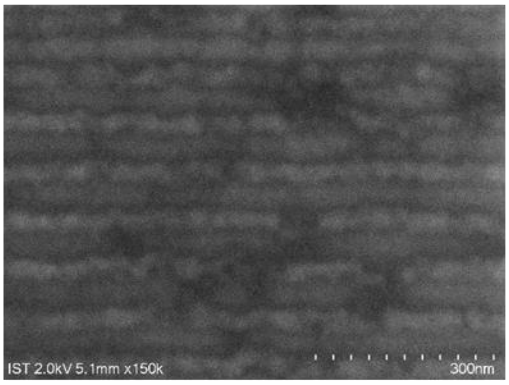
		**Dose = 1120 $\mu C/cm^2$**
**Design HP = 30 nm**		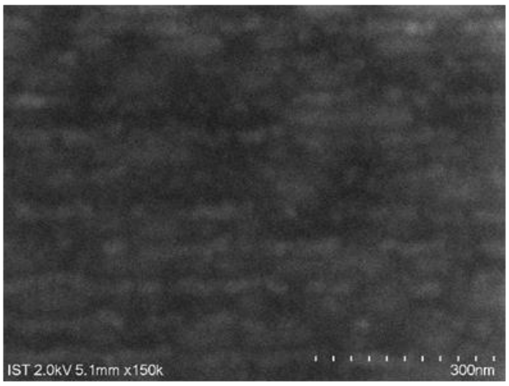

HP \ Dose	Dose = 1440 μC/cm^2, HP = 52 nm	Dose = 2440 μC/cm^2, HP = 51 nm
**Design HP = 50 nm**	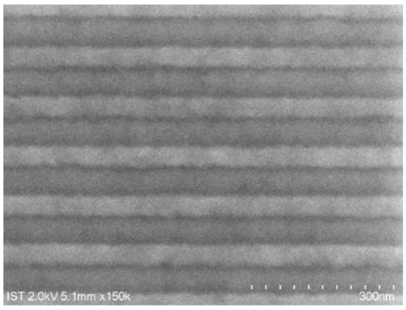	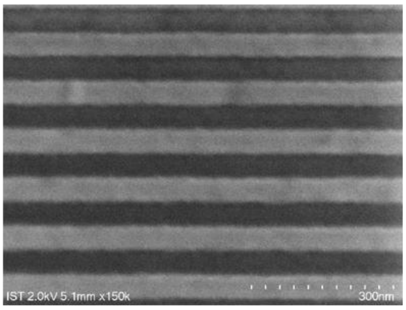
	**Dose = 1440 μC/cm^2, HP = 41 nm**	**Dose = 2440 μC/cm^2, HP = 42 nm**
**Design HP = 40 nm**	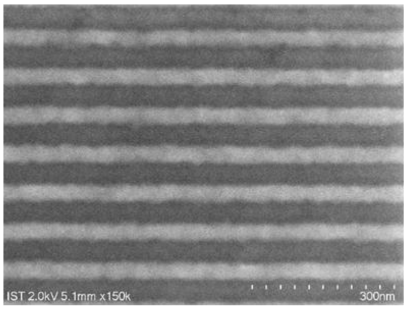	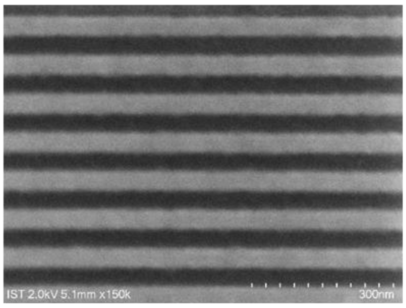
	**Dose = 1440 μC/cm^2, HP = 30 nm**	**Dose = 2440 μC/cm^2, HP = 31 nm**
**Design HP = 30 nm**	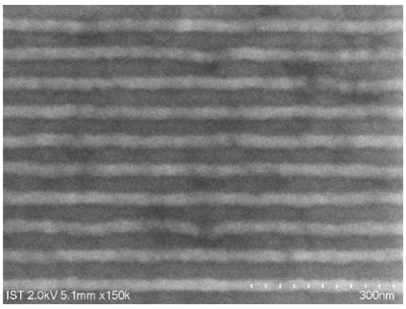	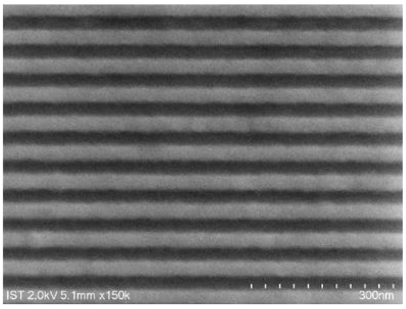
	**Dose = 1440 μC/cm^2**	**Dose = 2440 μC/cm^2, HP = 20 nm**
**Design HP = 20 nm**	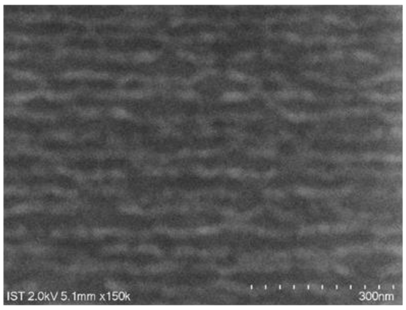	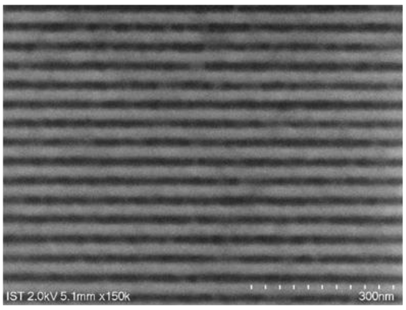

Figure S2. SEM images of E-beam lithography patterns on photoresist 3. Process parameter: 1.5 wt%, thickness 22.3 nm, Developer: 2-heptanone 60 s, PEB =80 ℃, 60 s. (Dose = 800-2400 μC/cm^2).

(3). SEM images of the EUV lithographic patterns

<table>
<tr><th colspan="2">PAB = 80 ℃ 60 s, PEB = 160 ℃ 60 s</th></tr>
<tr><td>HP = 16 nm, Dose = 207 mJ/cm^2</td><td>HP = 16 nm, Dose = 261 mJ/cm^2</td></tr>
<tr><td>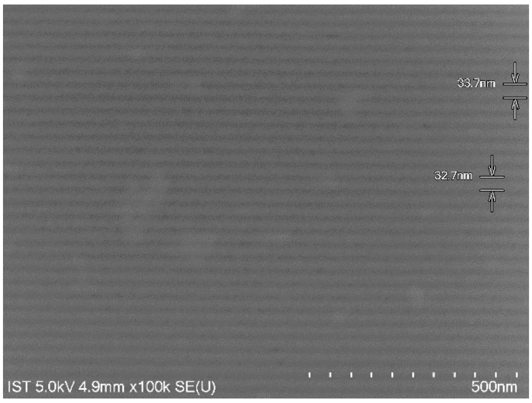
</td><td>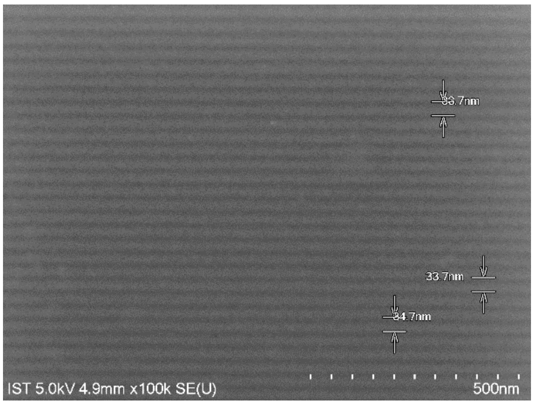
</td></tr>
<tr><td>HP = 15 nm, Dose = 205 mJ/cm^2</td><td>HP = 15 nm, Dose = 258 mJ/cm^2</td></tr>
<tr><td>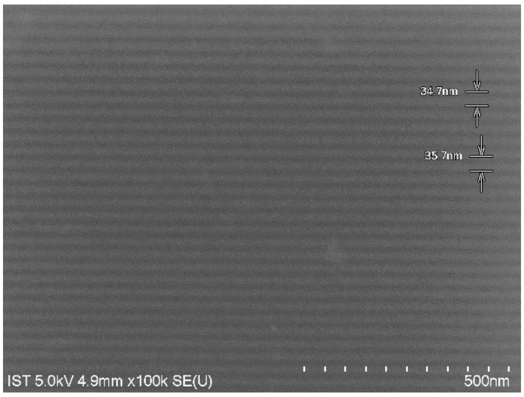
</td><td>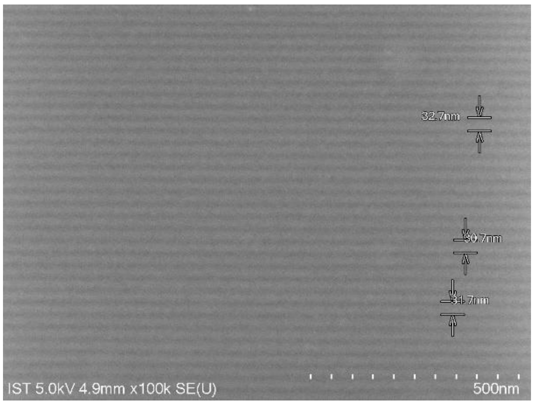
</td></tr>
<tr><td>HP = 14 nm, Dose = 216 mJ/cm^2</td><td>HP = 14 nm, Dose = 273 mJ/cm^2</td></tr>
<tr><td>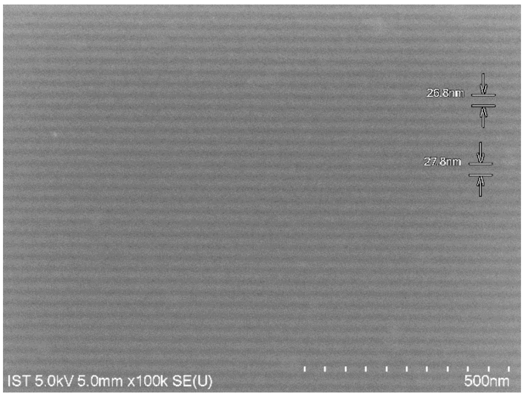
</td><td>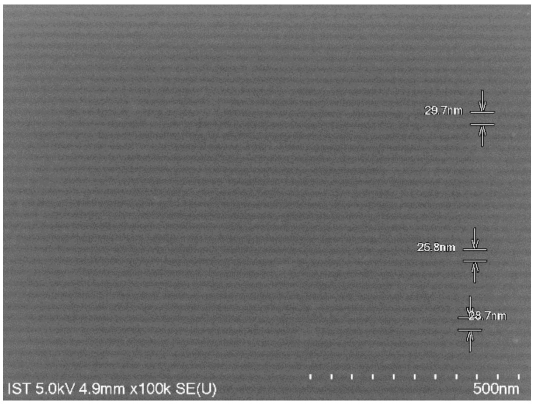
</td></tr>
<tr><td>HP = 13 nm, Dose = 205 mJ/cm^2</td><td>HP = 14 nm, Dose = 260mJ/cm^2</td></tr>
</table>

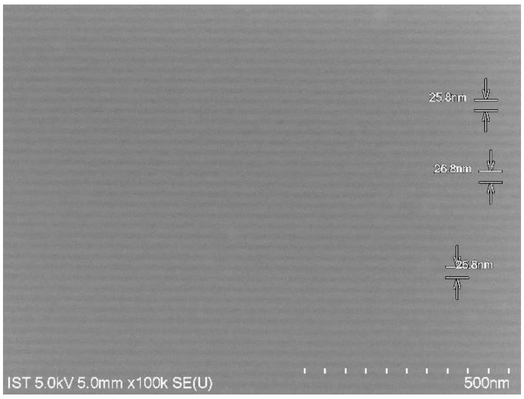

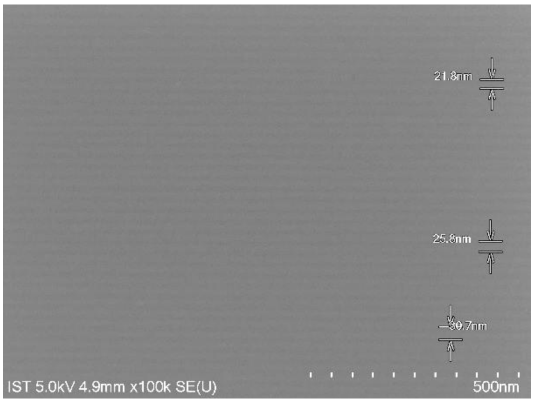

HP = 12 nm, Dose = 216 mJ/cm^2	HP = 12 nm, Dose = 268 mJ/cm^2

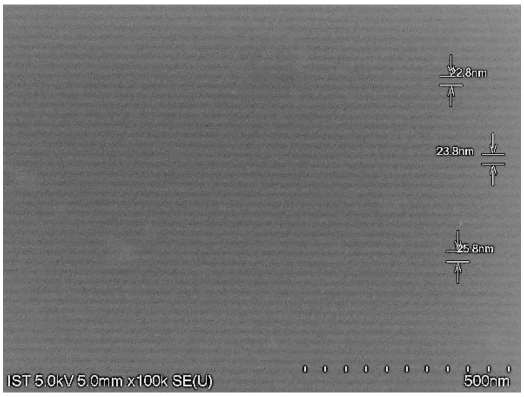

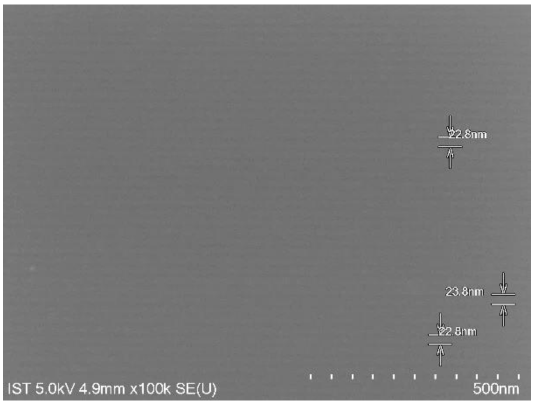

HP = 11 nm, Dose = 138 mJ/cm^2	HP = 11 nm, Dose = 174 mJ/cm^2

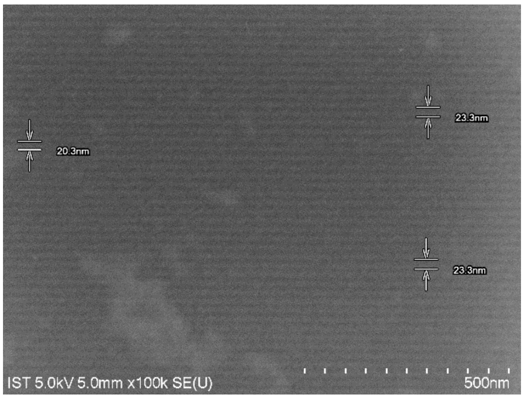

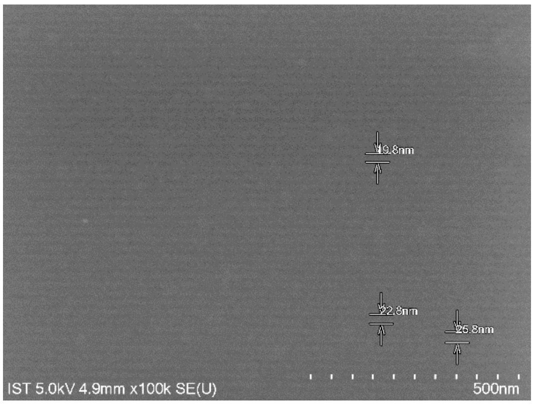

Figure S3. SEM images of EUV lithography patterns on photoresist (**2'**) HP= 11-16 nm at different dose. Process parameter: 1.5 wt%, thickness 25.0 nm, Developer: 2-Heptanone 60 s, PEB= 160°C, 60 s.

Dose / HP	Dose = 77 mJ/cm^2, HP = 50 nm	Dose = 88 mJ/cm^2 HP = 50 nm
**Design HP = 50 nm**	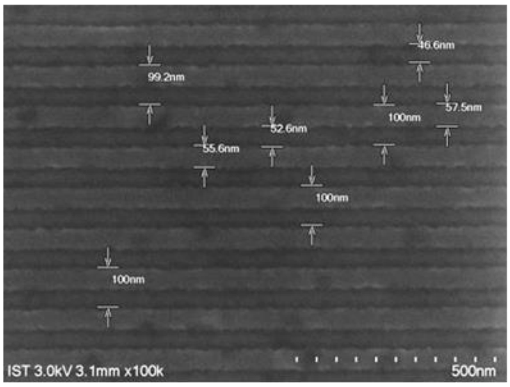	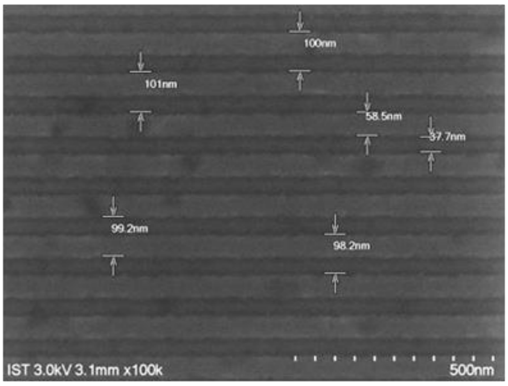
	**Dose = 70 mJ/cm^2, HP = 35 nm**	**Dose = 80 mJ/cm^2 HP = 35 nm**
**Design HP = 35 nm**	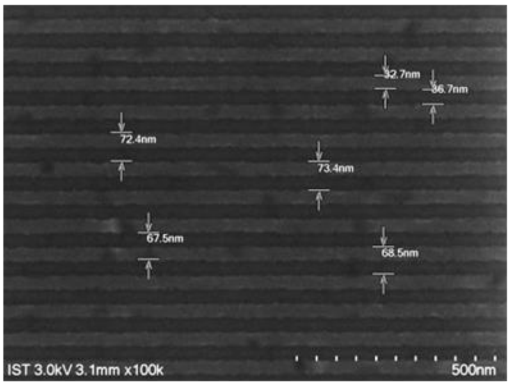	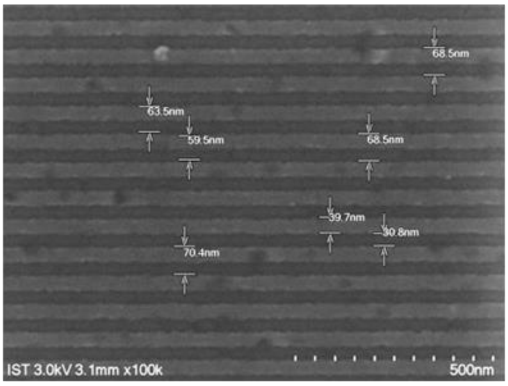
	**Dose = 64 mJ/cm^2, HP = 25 nm**	**Dose = 66 mJ/cm^2 HP = 25 nm**
**Design HP = 25 nm**	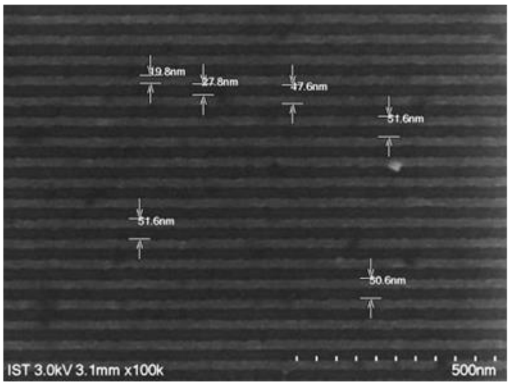	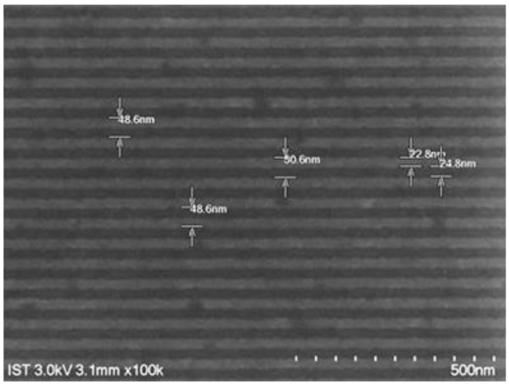

HP \ Dose	Dose = 63 mJ/cm^2, HP = 22 nm	Dose = 66 mJ/cm^2, HP = 22 nm
Design HP = 22 nm	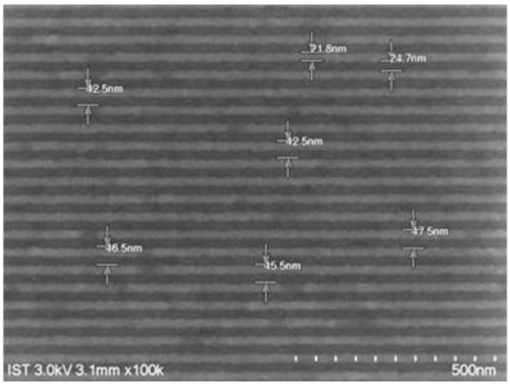	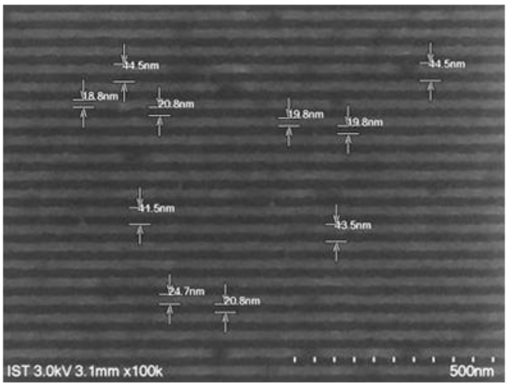
	Dose = 69 mJ/cm^2, HP = 18 nm	Dose = 66 mJ/cm^2
Design HP = 18 nm	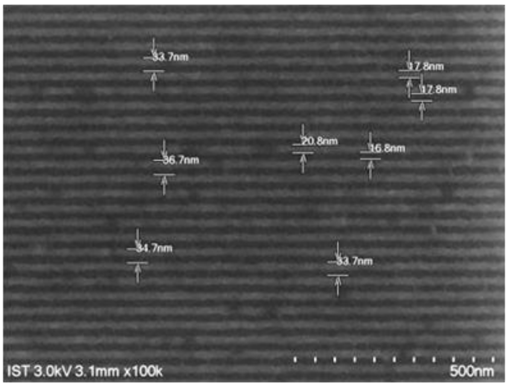	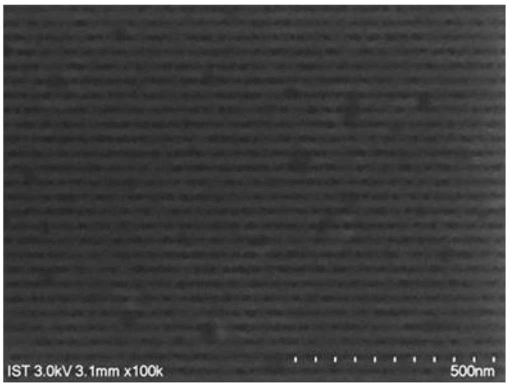
	Dose = 70 mJ/cm^2, HP = 16 nm	Dose = 66mJ/cm^2
Design HP = 16 nm	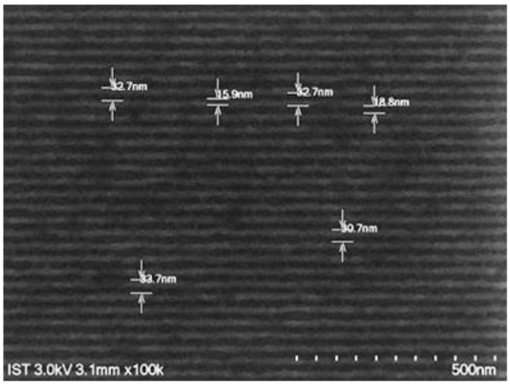	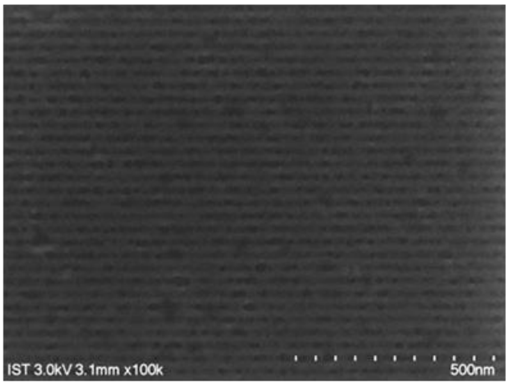

Dose / HP	Dose = 66 mJ/cm^2, HP = 51 nm	Dose = 60 mJ/cm^2, HP = 35 nm	Dose / HP
Design HP = 50 nm	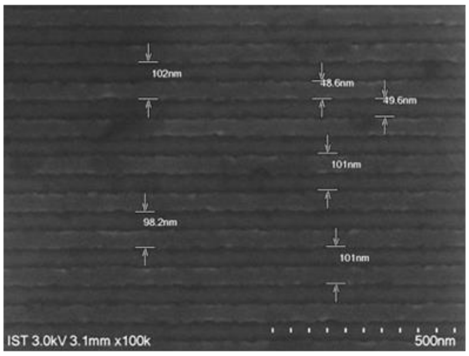	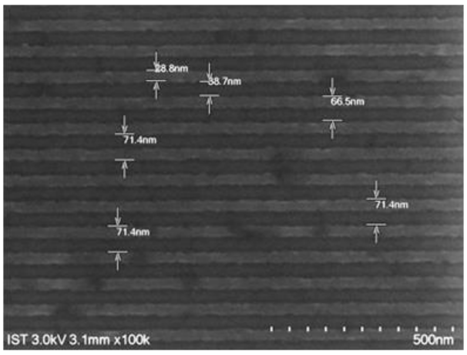	Design HP = 35 nm
	Dose = 55 mJ/cm^2, HP = 26 nm	Dose = 54 mJ/cm^2, HP = 22 nm	
Design HP = 25 nm	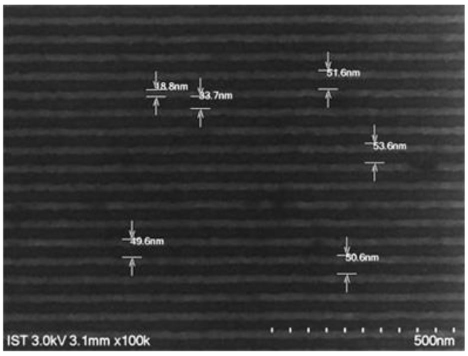	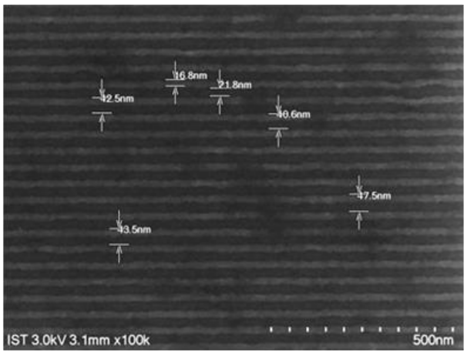	Design HP = 22 nm

Dose / HP	Dose = 82 mJ/cm^2, HP = 26 nm	Dose = 82 mJ/cm^2, HP = 23 nm	Dose / HP
Design HP = 25 nm	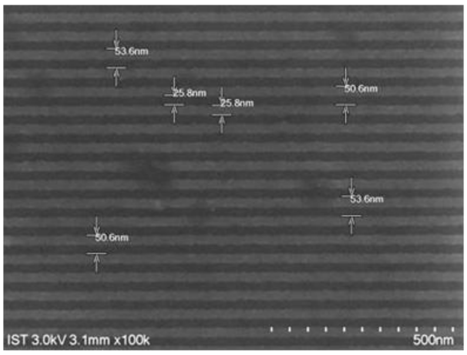	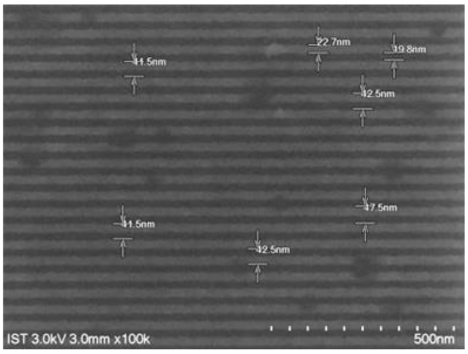	Design HP = 22 nm
	Dose = 89 mJ/cm^2, HP = 19 nm	Dose = 90 mJ/cm^2, HP = 17 nm	
Design HP = 18 nm	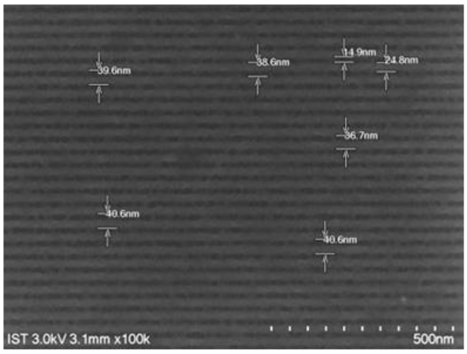	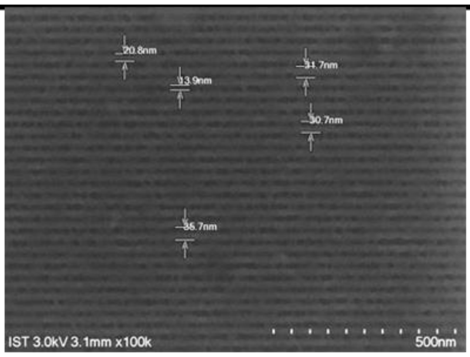	Design HP = 16 nm

Figure S4. SEM images of EUV lithography patterns on photoresist **3** with HP= 50, 35, 25, 22, 18, 16 nm at different dose. Process parameter: 1.5 wt%, thickness 22.3 nm, Developer: 2-Heptanone 60 s, PEB= 80°C, 60 s.(dose = 60-90 mJ/cm^2).

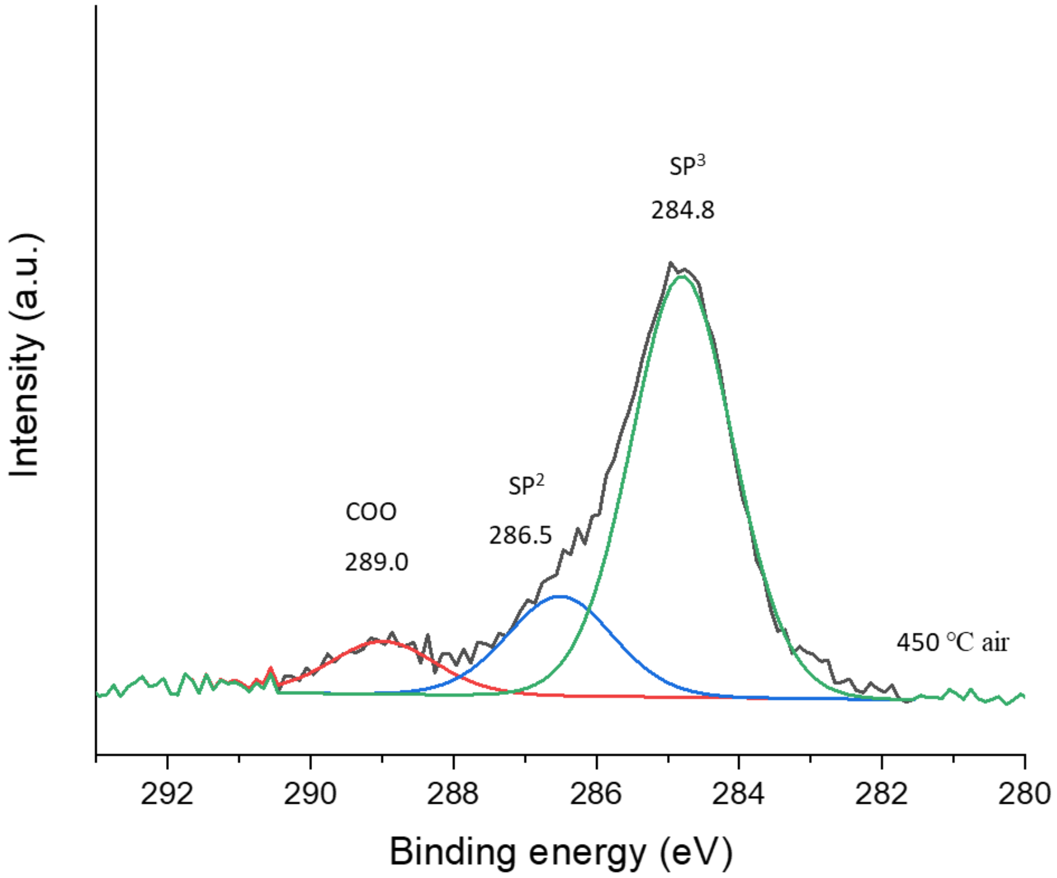

Figure s5.. Band-shape fitting spectra of the C\(1s) component of the TGA residues after heating at 450 ^{0}C.

4. Spectral data of key compounds.

cluster 1:

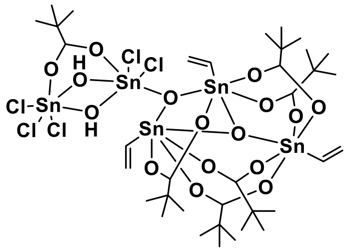

Cluster 1 was purified on recrystallization using DCM/hexane in low temperature. ^{1}H NMR (400 MHz, $CDCl_3$): δ 6.14-6.57 (m, 3 H), 1.22 (s, 18H); ^{13}C NMR (125 MHz, $CDCl_3$): δ 184.8, 139.6, 134.2, 123.0, 38.5, 29.7, 27.3, 27.2, 27.0; ^{119}Sn NMR (186 MHz, $CDCl_3$): δ -32.2. EA. calc. for $C_{36}H_{65}Cl_5O_{16}Sn_5$. C, 28.36%; H, 4.30%; O, 16.79%. found: C, 29.39%; H, 4.42%; O, 16.89%.

5. X-ray crystallographic data of cluster 1.

Ellipsoid contour % probability level = 50%

Experimental: The sample was dissolved in appropriate amount of DCM followed by the addition of *n*-hexane to furnish a saturated solution. Afterwards, the mixture was allowed to stand at low temperature (-4 ℃) to form the crystals.

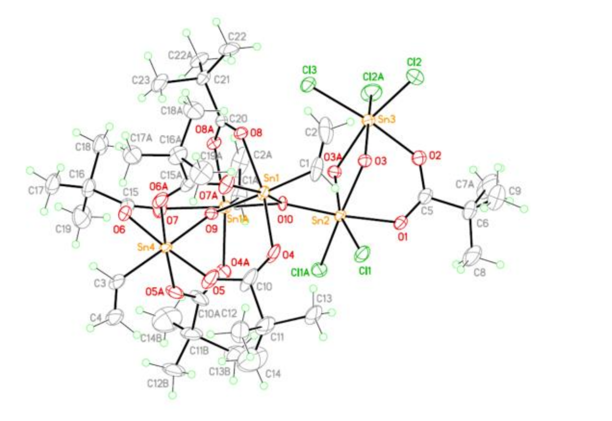

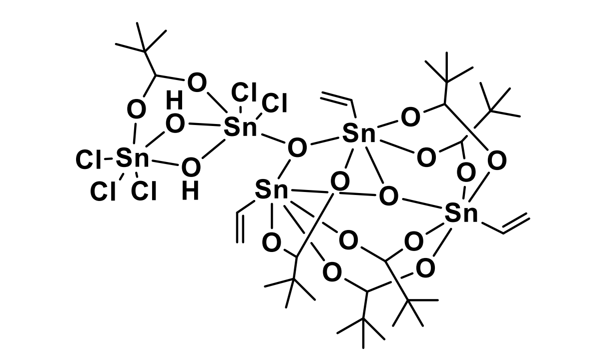

CCDC: **2300897**

ORTEP diagram of cluster 1

Table s1 Crystal data and structure refinement for 221175lt_auto.

Identification code	221175lt_auto
Empirical formula	$C_{36}H_{63}Cl_5O_{16}Sn_5$
Formula weight	1522.56
Temperature/K	100.01(10)
Crystal system	monoclinic
Space group	$P2_1/m$
a/Å	11.92010(10)
b/Å	16.4662(2)
c/Å	14.64400(10)
α/°	90
β/°	103.9010(10)
γ/°	90
Volume/Å^3	2790.12(5)
Z	2
ρ_{calc}g/cm^3	1.812
μ/mm^{-1}	20.239
F(000)	1484.0
Crystal size/mm^3	0.12 × 0.1 × 0.05
Radiation	Cu Kα (λ = 1.54184)
2Θ range for data collection/°	6.218 to 134.146
Index ranges	-14 ≤ h ≤ 14, -19 ≤ k ≤ 19, -17 ≤ l ≤ 11

Reflections collected	20553
Independent reflections	5167 [R_{int} = 0.0471, R_{sigma} = 0.0272]
Data/restraints/parameters	5167/258/406
Goodness-of-fit on F^2	1.090
Final R indexes [I>=2σ (I)]	R_1 = 0.0595, wR_2 = 0.1573
Final R indexes [all data]	R_1 = 0.0608, wR_2 = 0.1584
Largest diff. peak/hole / e Å^{-3}	2.60/-2.47

Table s2 Fractional Atomic Coordinates (×10^4) and Equivalent Isotropic Displacement Parameters (Å^2×10^3) for 221175lt_auto. U_{eq} is defined as 1/3 of the trace of the orthogonalised U_{IJ} tensor.

Atom	*x*	*y*	*z*	U(eq)
C1	3684(11)	2500	2330(9)	48(4)
C2	2491(17)	2500	1684(14)	77(5)
C3	1570(14)	2500	2180(13)	78(5)
C5	7208(8)	4456(6)	5213(7)	47(2)
C6	7754(9)	4674(7)	4581(8)	59(3)
C7	9607(9)	2500	5503(7)	27(2)
C8	10773(9)	2500	5277(8)	36(3)
C9	10950(8)	1732(7)	4736(7)	48(2)
C10	11663(11)	2500	6248(10)	64(5)
C11	9704(7)	4040(6)	7825(6)	39(2)
C16	9062(12)	2500	9896(9)	51(3)
C17	8475(12)	2500	10440(9)	58(4)
C18	6524(9)	3745(11)	7783(7)	81(5)
Cl1	4412.3(17)	1413.4(15)	4988.0(14)	40.1(5)
Cl2	8319(2)	2500	3206.0(19)	42.3(7)
Cl3	6417(2)	1391.3(18)	1447.8(16)	52.8(6)
O1	3776(7)	2500	3203(5)	37(2)
O2	4518(7)	2500	1914(5)	43(2)
O3	9174(4)	3177(4)	5619(4)	29.9(12)
O4	8880(5)	4183(4)	7136(4)	40.2(14)

Table s2 Fractional Atomic Coordinates (×10^4) and Equivalent Isotropic Displacement Parameters (Å^2×10^3) for 221175lt_auto. U_{eq} is defined as 1/3 of the trace of the orthogonalised U_{IJ} tensor.

Atom	*x*	*y*	*z*	U(eq)
O5	9825(5)	3403(4)	8320(4)	39.6(15)
O6	7298(6)	3402(6)	8376(4)	68(3)
O7	6456(5)	3760(5)	6903(4)	49.3(18)
O8	6772(6)	2500	5381(5)	27.9(17)
O9	8168(6)	2500	7003(5)	25.9(16)
O10	5918(4)	1728(4)	3478(3)	30.7(12)
Sn1	7677.8(4)	3470.7(3)	6125.4(3)	28.09(19)
Sn2	5248.4(6)	2500	4354.1(4)	26.9(2)
Sn3	6322.1(6)	2500	2446.3(5)	29.8(2)
Sn4	8583.9(6)	2500	8410.9(5)	37.4(3)
C12	10627(17)	4630(15)	8207(14)	45(3)
C13	10471(19)	5321(14)	7494(15)	62(4)
C14	11810(15)	4252(14)	8370(18)	55(4)
C15	10471(18)	4942(14)	9148(14)	55(4)
C19	5840(16)	4543(15)	8001(14)	60(4)
C20	6350(20)	5368(18)	7910(20)	88(7)
C21	5670(20)	4290(20)	9048(15)	64(5)
C22	4500(20)	4300(20)	7430(20)	70(6)
C12A	10664(19)	4749(14)	8008(14)	45(3)
C13A	11147(18)	4859(14)	7131(13)	49(4)
C14A	11628(17)	4529(15)	8832(14)	49(4)
C15A	10094(19)	5545(14)	8161(16)	57(4)
C19A	5415(18)	3922(15)	8175(15)	66(4)
C20A	4830(30)	3200(19)	8470(30)	100(7)
C21A	6020(30)	4570(20)	9018(17)	68(6)
C22A	4740(20)	4619(18)	7410(20)	64(6)
C4A	2410(19)	2190(20)	743(16)	96(11)
C4	2450(30)	1410(30)	1470(30)	110(12)

Table s3 Anisotropic Displacement Parameters (Å^2×10^3) for 221175lt_auto. The Anisotropic displacement factor exponent takes the form: $-2\pi^2[h^2a^{*2}U_{11}+2hka^*b^*U_{12}+…]$.

Atom	U_{11}	U_{22}	U_{33}	U_{23}	U_{13}	U_{12}
C1	23(6)	96(12)	23(6)	0	2(5)	0
C2	57(8)	117(10)	56(8)	0	9(7)	0
C3	33(8)	133(14)	59(10)	0	-3(7)	0
C5	30(5)	50(6)	49(6)	-3(5)	-14(4)	8(4)
C6	43(6)	55(6)	64(7)	25(5)	-18(5)	-12(5)
C7	24(5)	40(6)	12(4)	0	-4(4)	0
C8	15(5)	74(9)	18(5)	0	0(4)	0
C9	29(5)	74(7)	44(5)	12(5)	18(4)	18(5)
C10	17(6)	136(16)	32(7)	0	-7(5)	0
C11	23(4)	65(6)	26(4)	-16(4)	1(3)	9(4)
C16	26(5)	96(8)	28(6)	0	-1(5)	0
C17	32(7)	121(12)	24(6)	0	11(5)	0
C18	32(5)	178(15)	27(5)	-40(7)	-7(4)	39(7)
Cl1	23.8(9)	66.8(14)	28.0(10)	7.9(9)	2.8(8)	-7.8(9)
Cl2	23.5(13)	80(2)	20.6(13)	0	0.8(10)	0
Cl3	41.3(12)	85.2(18)	31.6(11)	-17.8(11)	8.4(9)	-2.2(12)
O1	20(4)	70(6)	17(4)	0	-1(3)	0
O2	23(4)	88(7)	14(4)	0	-5(3)	0
O3	21(3)	50(3)	18(2)	4(2)	3(2)	1(2)
O4	25(3)	51(4)	37(3)	-14(3)	-7(3)	2(3)
O5	20(3)	74(5)	21(3)	-8(3)	-2(2)	4(3)
O6	33(4)	148(8)	19(3)	-24(4)	0(3)	28(4)
O7	22(3)	93(5)	26(3)	-22(3)	-7(2)	16(3)
O8	24(4)	48(5)	8(3)	0	-3(3)	0
O9	11(3)	51(5)	15(3)	0	2(3)	0
O10	20(3)	55(4)	15(2)	1(2)	1(2)	1(2)
Sn1	18.9(3)	46.2(4)	16.5(3)	-3.1(2)	-0.98(19)	3.5(2)
Sn2	15.0(3)	51.0(5)	13.2(3)	0	0.1(3)	0
Sn3	21.4(4)	53.5(5)	14.0(3)	0	3.2(3)	0
Sn4	13.1(3)	89.6(7)	8.4(3)	0	0.2(2)	0
C12	32(5)	61(7)	40(7)	-9(5)	3(5)	-11(6)
C13	50(8)	71(9)	56(8)	2(7)	-4(7)	-19(7)

Table s3 Anisotropic Displacement Parameters (Å^2×10^3) for 221175lt_auto. The Anisotropic displacement factor exponent takes the form: $-2\pi^2[h^2a^{*2}U_{11}+2hka^*b^*U_{12}+\ldots]$.

Atom	U_{11}	U_{22}	U_{33}	U_{23}	U_{13}	U_{12}
C14	26(7)	71(10)	61(10)	-14(8)	-1(7)	-13(6)
C15	48(8)	69(9)	46(7)	-13(7)	4(7)	-8(7)
C19	35(7)	100(11)	47(7)	-15(8)	12(6)	21(7)
C20	67(13)	100(14)	99(15)	-2(12)	26(12)	17(10)
C21	35(10)	116(14)	44(7)	-13(9)	15(7)	28(9)
C22	33(9)	116(15)	56(8)	-26(11)	0(8)	32(9)
C12A	33(6)	61(7)	37(7)	-9(6)	3(5)	-12(6)
C13A	45(8)	64(9)	37(7)	-4(7)	7(6)	-21(7)
C14A	34(7)	70(9)	38(7)	-1(7)	1(6)	-25(7)
C15A	55(8)	63(8)	51(9)	-8(7)	8(7)	-12(7)
C19A	37(8)	108(12)	55(7)	-20(8)	13(6)	24(7)
C20A	71(14)	122(16)	115(17)	-7(12)	35(13)	12(11)
C21A	55(12)	113(14)	38(8)	-14(9)	13(8)	30(9)
C22A	29(9)	109(14)	51(8)	-30(10)	1(8)	25(9)
C4A	36(10)	180(30)	56(12)	-44(14)	-24(9)	1(13)
C4	53(14)	170(30)	90(17)	-41(18)	-13(14)	-21(16)

Table s4 Bond Lengths for 221175lt_auto.

Atom	Atom	Length/Å	Atom	Atom	Length/Å
C1	C2	1.51(2)	O2	Sn3	2.104(8)
C1	O1	1.257(15)	O3	Sn1	2.145(5)
C1	O2	1.285(15)	O4	Sn1	2.145(6)
C2	C3	1.45(3)	O5	Sn4	2.124(7)
C2	C4A	1.45(3)	O6	Sn4	2.126(8)
C2	C4	1.82(4)	O7	Sn1	2.108(6)
C5	C6	1.304(16)	O8	Sn1[1]	2.084(4)
C5	Sn1	2.091(10)	O8	Sn1	2.084(4)
C7	C8	1.503(15)	O8	Sn2	2.061(7)
C7	O3[1]	1.258(8)	O9	Sn1[1]	2.047(4)
C7	O3	1.258(8)	O9	Sn1	2.047(4)
C8	C9[1]	1.534(13)	O9	Sn4	2.001(7)

Table s4 Bond Lengths for 221175lt_auto.

Atom	Atom	Length/Å	Atom	Atom	Length/Å
C8	C9	1.534(12)	O10	Sn2	2.095(6)
C8	C10	1.556(16)	O10	Sn3	2.116(6)
C11	O4	1.250(10)	Sn1	Sn1[1]	3.1967(11)
C11	O5	1.264(12)	C12	C13	1.53(2)
C11	C12	1.47(3)	C12	C14	1.51(2)
C11	C12A	1.61(3)	C12	C15	1.52(2)
C16	C17	1.18(2)	C19	C20	1.51(3)
C16	Sn4	2.112(13)	C19	C21	1.65(2)
C18	O6	1.240(14)	C19	C22	1.66(2)
C18	O7	1.272(12)	C12A	C13A	1.54(2)
C18	C19	1.62(2)	C12A	C14A	1.50(2)
C18	C19A	1.59(2)	C12A	C15A	1.52(2)
Cl1	Sn2	2.345(2)	C19A	C20A	1.49(3)
Cl2	Sn3	2.373(3)	C19A	C21A	1.66(2)
Cl3	Sn3	2.359(3)	C19A	C22A	1.67(3)
O1	Sn2	2.121(7)			

[1]+X,1/2-Y,+Z

Table s5 Bond Angles for 221175lt_auto.

Atom	Atom	Atom	Angle/°	Atom	Atom	Atom	Angle/°
O1	C1	C2	118.5(13)	O8	Sn2	Cl1	95.45(14)
O1	C1	O2	126.5(11)	O8	Sn2	Cl1[1]	95.45(14)
O2	C1	C2	115.0(12)	O8	Sn2	O1	174.6(3)
C1	C2	C4	95.2(12)	O8	Sn2	O10[1]	92.8(2)
C3	C2	C1	113.4(15)	O8	Sn2	O10	92.8(2)
C3	C2	C4	95.1(15)	O10	Sn2	Cl1	92.30(17)
C4A	C2	C1	115.8(16)	O10[1]	Sn2	Cl1	164.94(16)
C4A	C2	C3	126.1(17)	O10	Sn2	Cl1[1]	164.94(16)
C6	C5	Sn1	124.3(8)	O10[1]	Sn2	Cl1[1]	92.31(17)
O3	C7	C8	117.4(5)	O10[1]	Sn2	O1	83.0(2)
O3[1]	C7	C8	117.4(5)	O10	Sn2	O1	83.0(2)

Table s5 Bond Angles for 221175lt_auto.

Atom	Atom	Atom	Angle/°	Atom	Atom	Atom	Angle/°
O3	C7	O3[1]	125.0(10)	O10[1]	Sn2	O10	74.7(3)
C7	C8	C9[1]	111.3(6)	Cl3[1]	Sn3	Cl2	95.65(8)
C7	C8	C9	111.3(6)	Cl3	Sn3	Cl2	95.65(8)
C7	C8	C10	105.2(9)	Cl3	Sn3	Cl3[1]	101.43(14)
C9	C8	C9[1]	111.1(10)	O2	Sn3	Cl2	174.0(2)
C9	C8	C10	108.9(7)	O2	Sn3	Cl3[1]	88.13(15)
C9[1]	C8	C10	108.9(7)	O2	Sn3	Cl3	88.13(15)
O4	C11	O5	125.4(9)	O2	Sn3	O10[1]	82.9(2)
O4	C11	C12	123.6(12)	O2	Sn3	O10	82.9(2)
O4	C11	C12A	112.8(11)	O10[1]	Sn3	Cl2	92.30(15)
O5	C11	C12	110.9(11)	O10	Sn3	Cl2	92.30(15)
O5	C11	C12A	121.8(9)	O10	Sn3	Cl3[1]	163.82(17)
C17	C16	Sn4	129.7(12)	O10	Sn3	Cl3	91.76(17)
O6	C18	O7	125.0(9)	O10[1]	Sn3	Cl3[1]	91.77(17)
O6	C18	C19	123.9(11)	O10[1]	Sn3	Cl3	163.81(17)
O6	C18	C19A	112.0(12)	O10	Sn3	O10[1]	73.8(3)
O7	C18	C19	105.8(13)	C16	Sn4	O5[1]	92.6(3)
O7	C18	C19A	120.4(11)	C16	Sn4	O5	92.6(3)
C1	O1	Sn2	131.4(8)	C16	Sn4	O6[1]	92.3(3)
C1	O2	Sn3	131.5(7)	C16	Sn4	O6	92.3(3)
C7	O3	Sn1	130.5(6)	O5[1]	Sn4	O5	88.9(3)
C11	O4	Sn1	136.0(7)	O5	Sn4	O6[1]	175.2(2)
C11	O5	Sn4	129.2(5)	O5	Sn4	O6	91.0(3)
C18	O6	Sn4	138.2(6)	O5[1]	Sn4	O6[1]	91.0(3)
C18	O7	Sn1	131.2(6)	O5[1]	Sn4	O6	175.2(2)
Sn1[1]	O8	Sn1	100.1(3)	O6	Sn4	O6[1]	88.6(5)
Sn2	O8	Sn1[1]	129.55(15)	O9	Sn4	C16	178.7(4)
Sn2	O8	Sn1	129.55(15)	O9	Sn4	O5[1]	86.5(2)
Sn1[1]	O9	Sn1	102.7(3)	O9	Sn4	O5	86.5(2)
Sn4	O9	Sn1	127.58(16)	O9	Sn4	O6	88.7(2)
Sn4	O9	Sn1[1]	127.58(16)	O9	Sn4	O6[1]	88.7(2)
Sn2	O10	Sn3	105.0(3)	C11	C12	C13	105.9(15)
C5	Sn1	O3	94.1(3)	C11	C12	C14	111.9(17)

Table s5 Bond Angles for 221175lt_auto.

Atom	Atom	Atom	Angle/°	Atom	Atom	Atom	Angle/°
C5	Sn1	O4	92.8(3)	C11	C12	C15	108.9(15)
C5	Sn1	O7	93.2(4)	C14	C12	C13	111.9(18)
C5	Sn1	Sn1[1]	140.9(3)	C14	C12	C15	108.0(17)
O3	Sn1	Sn1[1]	76.98(16)	C15	C12	C13	110.2(18)
O4	Sn1	O3	83.6(2)	C18	C19	C21	98.8(18)
O4	Sn1	Sn1[1]	123.15(17)	C18	C19	C22	99.9(18)
O7	Sn1	O3	167.9(2)	C20	C19	C18	118.9(17)
O7	Sn1	O4	86.4(2)	C20	C19	C21	117(2)
O7	Sn1	Sn1[1]	103.0(2)	C20	C19	C22	122(2)
O8	Sn1	C5	103.6(3)	C21	C19	C22	95.2(16)
O8	Sn1	O3	90.9(3)	C13A	C12A	C11	109.8(15)
O8	Sn1	O4	163.1(2)	C14A	C12A	C11	109.7(16)
O8	Sn1	O7	96.7(3)	C14A	C12A	C13A	109.4(17)
O8	Sn1	Sn1[1]	39.93(14)	C14A	C12A	C15A	111.9(19)
O9	Sn1	C5	178.7(4)	C15A	C12A	C11	109.0(16)
O9	Sn1	O3	84.6(2)	C15A	C12A	C13A	107.0(17)
O9	Sn1	O4	87.2(2)	C18	C19A	C21A	97.7(17)
O9	Sn1	O7	88.1(3)	C18	C19A	C22A	101.3(18)
O9	Sn1	O8	76.3(2)	C20A	C19A	C18	116(2)
O9	Sn1	Sn1[1]	38.65(15)	C20A	C19A	C21A	116(2)
Cl1[1]	Sn2	Cl1	99.44(12)	C20A	C19A	C22A	124(2)
O1	Sn2	Cl1[1]	88.00(15)	C21A	C19A	C22A	96.5(17)
O1	Sn2	Cl1	88.00(15)				

[1]+X,1/2-Y,+Z

Table s6 Torsion Angles for 221175lt_auto.

A	B	C	D	Angle/°	A	B	C	D	Angle/°
C2	C1	O1	Sn2	180.000(1)	O5	C11	C12	C14	51.4(18)
C2	C1	O2	Sn3	180.000(1)	O5	C11	C12	C15	-67.8(17)
C8	C7	O3	Sn1	-170.6(6)	O5	C11	C12A	C13A	118.7(14)
O1	C1	C2	C3	0.000(2)	O5	C11	C12A	C14A	-2(2)

Table s6 Torsion Angles for 221175lt_auto.

A	B	C	D	Angle/°	A	B	C	D	Angle/°
O1	C1	C2	C4A	-157.3(17)	O5	C11	C12A	C15A	-124.4(14)
O1	C1	C2	C4	-97.9(16)	O6	C18	O7	Sn1	-14(3)
O1	C1	O2	Sn3	0.000(2)	O6	C18	C19	C20	87(2)
O2	C1	C2	C3	180.000(1)	O6	C18	C19	C21	-41(2)
O2	C1	C2	C4A	22.7(17)	O6	C18	C19	C22	-138.1(19)
O2	C1	C2	C4	82.1(16)	O6	C18	C19A	C20A	-60(2)
O2	C1	O1	Sn2	0.000(2)	O6	C18	C19A	C21A	64(2)
O3[1]	C7	C8	C9	30.0(12)	O6	C18	C19A	C22A	162.6(16)
O3	C7	C8	C9	-154.4(8)	O7	C18	O6	Sn4	-18(3)
O3[1]	C7	C8	C9[1]	154.4(8)	O7	C18	C19	C20	-68(2)
O3	C7	C8	C9[1]	-30.0(12)	O7	C18	C19	C21	163.8(14)
O3	C7	C8	C10	87.8(8)	O7	C18	C19	C22	66.9(18)
O3[1]	C7	C8	C10	-87.8(8)	O7	C18	C19A	C20A	102(2)
O3[1]	C7	O3	Sn1	4.7(15)	O7	C18	C19A	C21A	-133.2(19)
O4	C11	O5	Sn4	-15.9(12)	O7	C18	C19A	C22A	-35(2)
O4	C11	C12	C13	-10.5(19)	C12	C11	O4	Sn1	158.5(10)
O4	C11	C12	C14	-132.7(16)	C12	C11	O5	Sn4	159.9(9)
O4	C11	C12	C15	108.1(16)	C19	C18	O6	Sn4	-168.2(12)
O4	C11	C12A	C13A	-58.9(16)	C19	C18	O7	Sn1	140.3(10)
O4	C11	C12A	C14A	-179.2(14)	C12A	C11	O4	Sn1	151.4(10)
O4	C11	C12A	C15A	57.9(17)	C12A	C11	O5	Sn4	166.7(10)
O5	C11	O4	Sn1	-26.2(14)	C19A	C18	O6	Sn4	143.7(13)
O5	C11	C12	C13	173.6(13)	C19A	C18	O7	Sn1	-174.5(13)

[1]+X,1/2-Y,+Z

Table s7 Hydrogen Atom Coordinates (Å×10^4) and Isotropic Displacement Parameters (Å^2×10^3) for 221175lt_auto.

Atom	*x*	*y*	*z*	U(eq)
H3A	1532.31	3033.25	2468.13	116
H3B	1725.88	2082.13	2670.58	116
H3C	831.18	2384.63	1734.84	116

Table s7 Hydrogen Atom Coordinates (Å×10^4) and Isotropic Displacement Parameters (Å^2×10^3) for 221175lt_auto.

Atom	*x*	*y*	*z*	U(eq)
H5	6552.26	4766.04	5262.66	56
H6A	8412.59	4377.93	4512.45	71
H6B	7493.19	5129.4	4188.41	71
H9A	10889.47	1251.87	5116.41	71
H9B	11716.81	1747.17	4602.38	71
H9C	10356.76	1706.91	4143.15	71
H10A	12427.16	2646.88	6161.6	96
H10B	11696.22	1957.56	6528.49	96
H10C	11424.81	2895.56	6664.72	96
H16	9870.11	2500	10166.93	61
H17A	7657.48	2500	10222.28	70
H17B	8820.52	2500	11096.28	70
H13A	10318.11	5096.08	6856.54	93
H13B	11175.71	5650.78	7612.51	93
H13C	9818.05	5661.3	7555.13	93
H14A	11865.25	3796.27	8808.86	82
H14B	12397.67	4658.73	8635.82	82
H14C	11935.65	4055.05	7770.73	82
H15A	9665.9	5106.81	9081.15	83
H15B	10980.44	5408.79	9347.48	83
H15C	10667.34	4509.8	9619.76	83
H20A	5757.31	5785.78	7892.54	132
H20B	6613.92	5390.81	7325.95	132
H20C	7001.97	5465.09	8447.43	132
H21A	6420.42	4319.51	9507.77	96
H21B	5379.56	3729.17	9026.31	96
H21C	5123.76	4657.01	9231.15	96
H22A	3974.08	4396.18	7837.62	105
H22B	4475.62	3724.43	7252.56	105
H22C	4269.97	4633.57	6861.87	105
H13D	11909.8	5116.61	7312.35	74
H13E	10622.39	5203.02	6673.25	74

Table s7 Hydrogen Atom Coordinates (Å×10^4) and Isotropic Displacement Parameters (Å^2×10^3) for 221175lt_auto.

Atom	*x*	*y*	*z*	U(eq)
H13F	11217.76	4327.37	6848.86	74
H14D	11972.9	4013.46	8707.03	73
H14E	11324.76	4474.36	9394.78	73
H14F	12217.45	4956.57	8934.72	73
H15D	10673.77	5979.02	8277.51	86
H15E	9753.61	5494.28	8704.65	86
H15F	9486.55	5676.33	7599.89	86
H20D	5405.41	2852.05	8878.85	150
H20E	4433.62	2892.97	7912.58	150
H20F	4263.22	3381.58	8814.29	150
H21D	5434.78	4781.52	9318.7	102
H21E	6374.28	5015.95	8745	102
H21F	6621.41	4285.48	9487.22	102
H22D	4338.03	4351.83	6824.41	96
H22E	5300.56	5007.59	7281.23	96
H22F	4169.81	4906.46	7679.03	96
H4AA	3178.85	2195.15	612.55	143
H4AB	1889.82	2541.07	285.93	143
H4AC	2107.2	1638.17	694.54	143
H4A	1781.68	1275.73	956.39	165
H4B	2373.51	1122.4	2044.68	165
H4C	3159.62	1235.94	1308.39	165

Table s8 Atomic Occupancy for 221175lt_auto.

Atom	*Occupancy*	Atom	*Occupancy*	Atom	*Occupancy*
H3A	0.5	H3B	0.5	H3C	0.5
H10A	0.5	H10B	0.5	H10C	0.5
C12	0.510(13)	C13	0.510(13)	H13A	0.510(13)
H13B	0.510(13)	H13C	0.510(13)	C14	0.510(13)
H14A	0.510(13)	H14B	0.510(13)	H14C	0.510(13)
C15	0.510(13)	H15A	0.510(13)	H15B	0.510(13)

Table s8 Atomic Occupancy for 221175lt_auto.

Atom	*Occupancy*	Atom	*Occupancy*	Atom	*Occupancy*
H15C	0.510(13)	C19	0.511(14)	C20	0.511(14)
H20A	0.511(14)	H20B	0.511(14)	H20C	0.511(14)
C21	0.511(14)	H21A	0.511(14)	H21B	0.511(14)
H21C	0.511(14)	C22	0.511(14)	H22A	0.511(14)
H22B	0.511(14)	H22C	0.511(14)	C12A	0.490(13)
C13A	0.490(13)	H13D	0.490(13)	H13E	0.490(13)
H13F	0.490(13)	C14A	0.490(13)	H14D	0.490(13)
H14E	0.490(13)	H14F	0.490(13)	C15A	0.490(13)
H15D	0.490(13)	H15E	0.490(13)	H15F	0.490(13)
C19A	0.489(14)	C20A	0.489(14)	H20D	0.489(14)
H20E	0.489(14)	H20F	0.489(14)	C21A	0.489(14)
H21D	0.489(14)	H21E	0.489(14)	H21F	0.489(14)
C22A	0.489(14)	H22D	0.489(14)	H22E	0.489(14)
H22F	0.489(14)	C4A	0.52(2)	H4AA	0.52(2)
H4AB	0.52(2)	H4AC	0.52(2)	C4	0.48(2)
H4A	0.48(2)	H4B	0.48(2)	H4C	0.48(2)

Experimental

Single crystals of $C_{36}H_{63}Cl_5O_{16}Sn_5$ **[221175lt_auto]** were **[]**. A suitable crystal was selected and **[]** on a **XtaLAB Synergy R, DW system, HyPix-Arc 150** diffractometer. The crystal was kept at 100.01(10) K during data collection. Using Olex2 [1], the structure was solved with the SHELXT [2] structure solution program using Intrinsic Phasing and refined with the SHELXL [3] refinement package using Least Squares minimisation.

1. Dolomanov, O.V., Bourhis, L.J., Gildea, R.J, Howard, J.A.K. & Puschmann, H. (2009), J. Appl. Cryst. 42, 339-341.
2. Sheldrick, G.M. (2015). Acta Cryst. A71, 3-8.
3. Sheldrick, G.M. (2015). Acta Cryst. C71, 3-8.

Crystal structure determination of [221175lt_auto]

Crystal Data for $C_{36}H_{63}Cl_5O_{16}Sn_5$ (M =1522.56 g/mol): monoclinic, space group P2_1/m (no. 11), a = 11.92010(10) Å, b = 16.4662(2) Å, c = 14.64400(10) Å, β = 103.9010(10)°, V = 2790.12(5) Å^3, Z = 2, T = 100.01(10) K, μ(Cu Kα) = 20.239 mm^{-1}, $Dcalc$ = 1.812 g/cm^3, 20553 reflections measured (6.218° ≤ 2Θ ≤ 134.146°), 5167 unique (R_{int} = 0.0471, R_{sigma} = 0.0272) which were used in all calculations. The final R_1 was 0.0595 (I > 2σ(I)) and wR_2 was 0.1584 (all data).

Refinement model description

Number of restraints - 258, number of constraints - unknown.

Details:

1. Fixed Uiso

At 1.2 times of:

All C(H) groups, All C(H,H) groups

At 1.5 times of:

All C(H,H,H) groups

2. Rigid bond restraints

C12, C13, C14, C15, C12A, C13A, C14A, C15A

with sigma for 1-2 distances of 0.01 and sigma for 1-3 distances of 0.01

C19, C20, C21, C22, C19A, C20A, C21A, C22A

with sigma for 1-2 distances of 0.01 and sigma for 1-3 distances of 0.01

C4, C4A

with sigma for 1-2 distances of 0.01 and sigma for 1-3 distances of 0.01

3. Uiso/Uaniso restraints and constraints

C12 ≈ C13 ≈ C14 ≈ C15 ≈ C12A ≈ C13A ≈ C14A ≈ C15A:

within 2A with sigma of 0.01 and sigma for terminal atoms of 0.02 within 2A

C19 ≈ C20 ≈ C21 ≈ C22 ≈ C19A ≈ C20A ≈ C21A ≈ C22A:

within 2A with sigma of 0.01 and sigma for terminal atoms of 0.02 within 2A

C4 ≈ C4A: within 2A with sigma of 0.01 and sigma for terminal atoms of 0.02 within 2A

Uanis(C2) ≈ Ueq, Uanis(C3) ≈ Ueq, Uanis(C7) ≈ Ueq, Uanis(C16) ≈ Ueq, Uanis(C17) ≈ Ueq: with sigma of 0.01 and sigma for terminal atoms of 0.02

4. Same fragment restrains

{C12, C13, C14, C15} sigma for 1-2: 0.02, 1-3: 0.04

as in

{C12A, C13A, C14A, C15A}

{C19, C20, C21, C22} sigma for 1-2: 0.02, 1-3: 0.04

as in

{C19A, C20A, C21A, C22A}

5. Others

Sof(C19A)=Sof(C20A)=Sof(H20D)=Sof(H20E)=Sof(H20F)=Sof(C21A)=Sof(H21D)=
Sof(H21E)=Sof(H21F)=Sof(C22A)=Sof(H22D)=Sof(H22E)=Sof(H22F)=1-FVAR(1)

Sof(C19)=Sof(C20)=Sof(H20A)=Sof(H20B)=Sof(H20C)=Sof(C21)=Sof(H21A)=Sof(H21B)=
Sof(H21C)=Sof(C22)=Sof(H22A)=Sof(H22B)=Sof(H22C)=FVAR(1)

Sof(C12A)=Sof(C13A)=Sof(H13D)=Sof(H13E)=Sof(H13F)=Sof(C14A)=Sof(H14D)=
Sof(H14E)=Sof(H14F)=Sof(C15A)=Sof(H15D)=Sof(H15E)=Sof(H15F)=1-FVAR(2)

Sof(C12)=Sof(C13)=Sof(H13A)=Sof(H13B)=Sof(H13C)=Sof(C14)=Sof(H14A)=Sof(H14B)=
Sof(H14C)=Sof(C15)=Sof(H15A)=Sof(H15B)=Sof(H15C)=FVAR(2)

Sof(C4A)=Sof(H4AA)=Sof(H4AB)=Sof(H4AC)=1-FVAR(3)

Sof(C4)=Sof(H4A)=Sof(H4B)=Sof(H4C)=FVAR(3)

Fixed Sof: H3A(0.5) H3B(0.5) H3C(0.5) H10A(0.5) H10B(0.5) H10C(0.5)

6.a Aromatic/amide H refined with riding coordinates:

C5(H5), C16(H16)

6.b X=CH2 refined with riding coordinates:

C6(H6A,H6B), C17(H17A,H17B)

6.c Idealised Me refined as rotating group:

C3(H3A,H3B,H3C), C9(H9A,H9B,H9C), C10(H10A,H10B,H10C), C13(H13A,H13B,H13C), C14(H14A,H14B,H14C), C15(H15A,H15B,H15C), C20(H20A,H20B,H20C), C21(H21A,H21B, H21C), C22(H22A,H22B,H22C), C13A(H13D,H13E,H13F), C14A(H14D,H14E,H14F), C15A(H15D,H15E,H15F), C20A(H20D,H20E,H20F), C21A(H21D,H21E,H21F), C22A(H22D, H22E,H22F), C4A(H4AA,H4AB,H4AC), C4(H4A,H4B,H4C)

This report has been created with Olex2, compiled on 2022.04.07 svn.rca3783a0 for OlexSys. Please let us know if there are any errors or if you would like to have additional features.

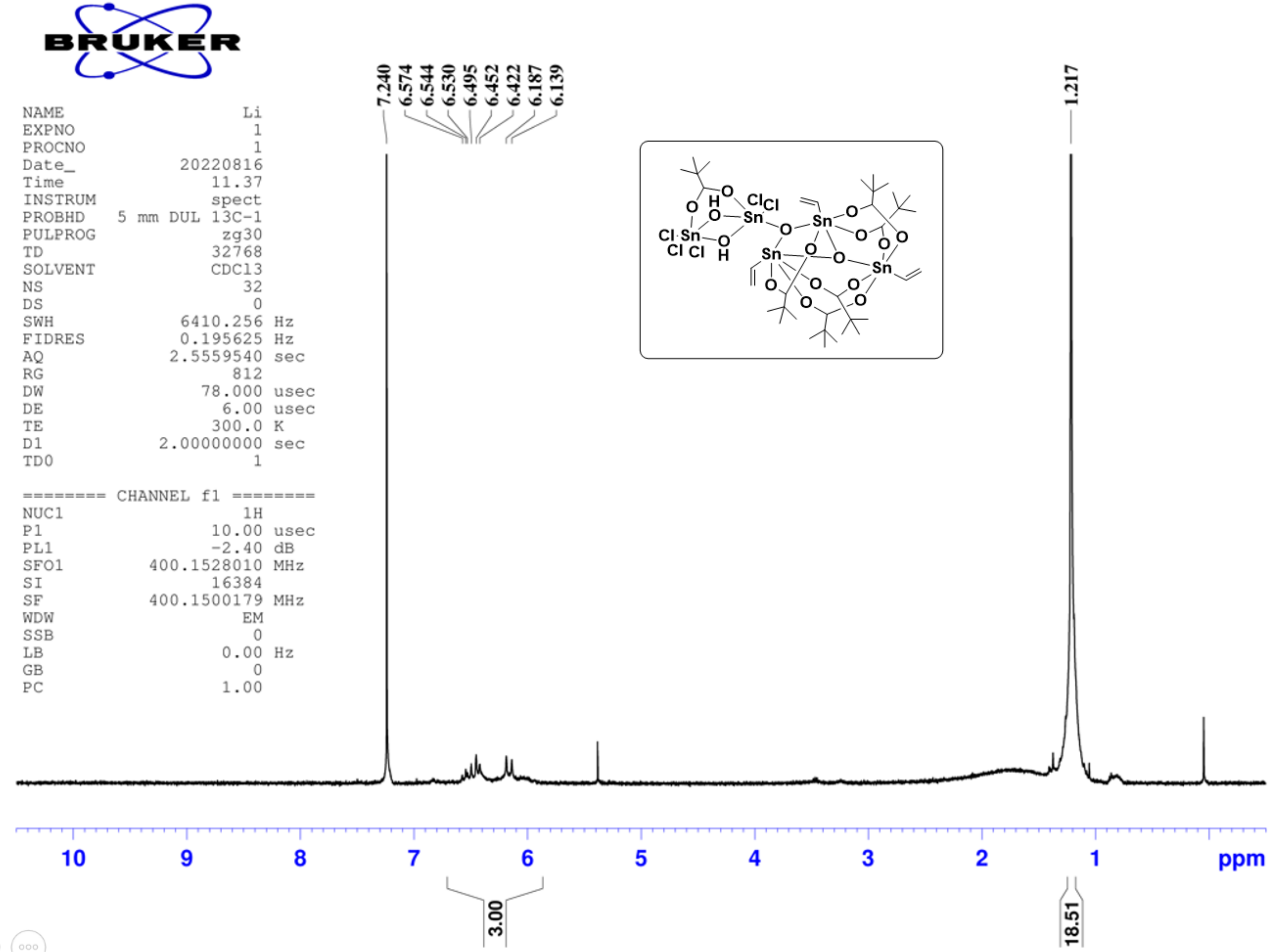

Figure S6. ^{1}H NMR of cluster 1

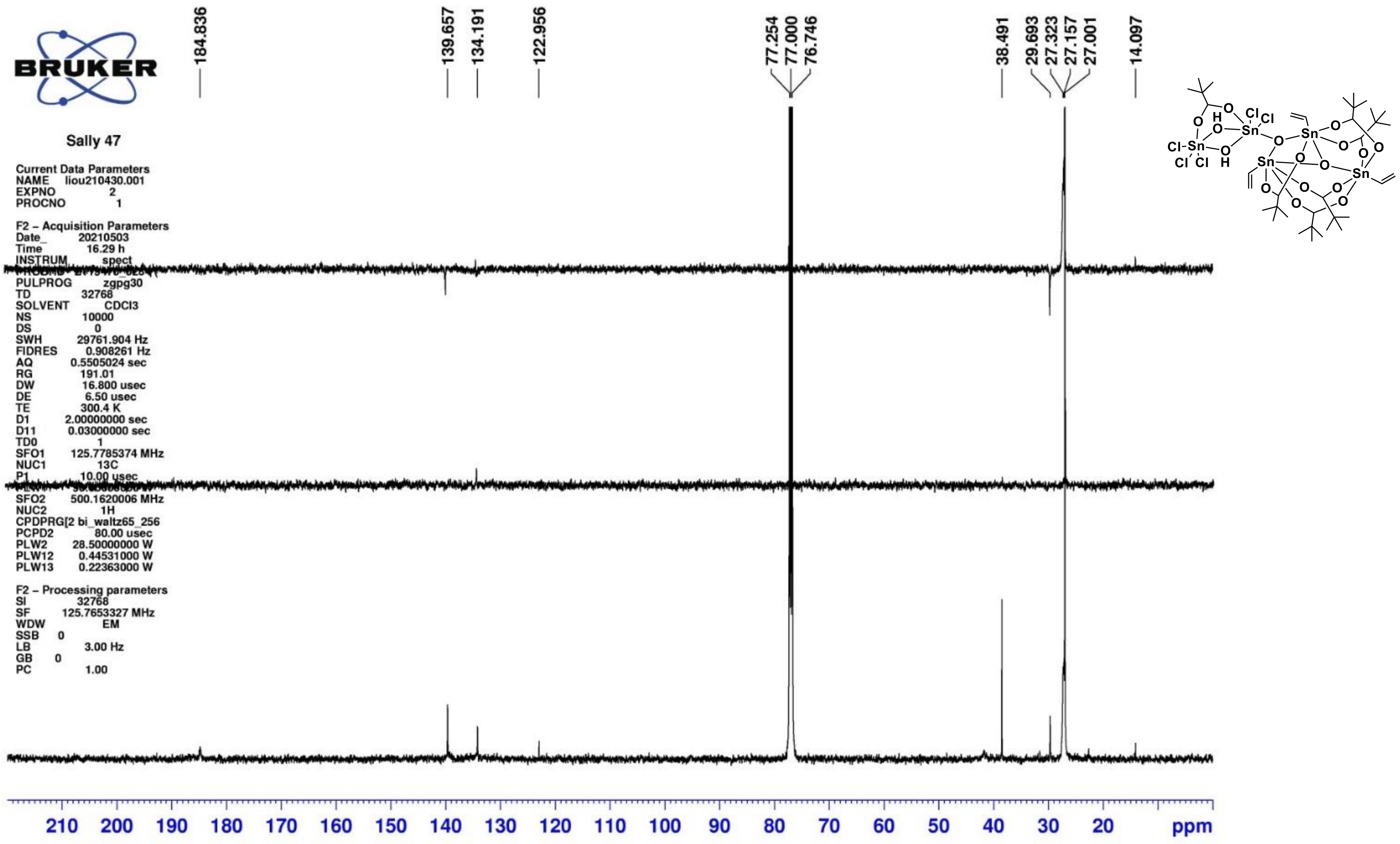

Figure S7. ^{13}C NMR of cluster 1

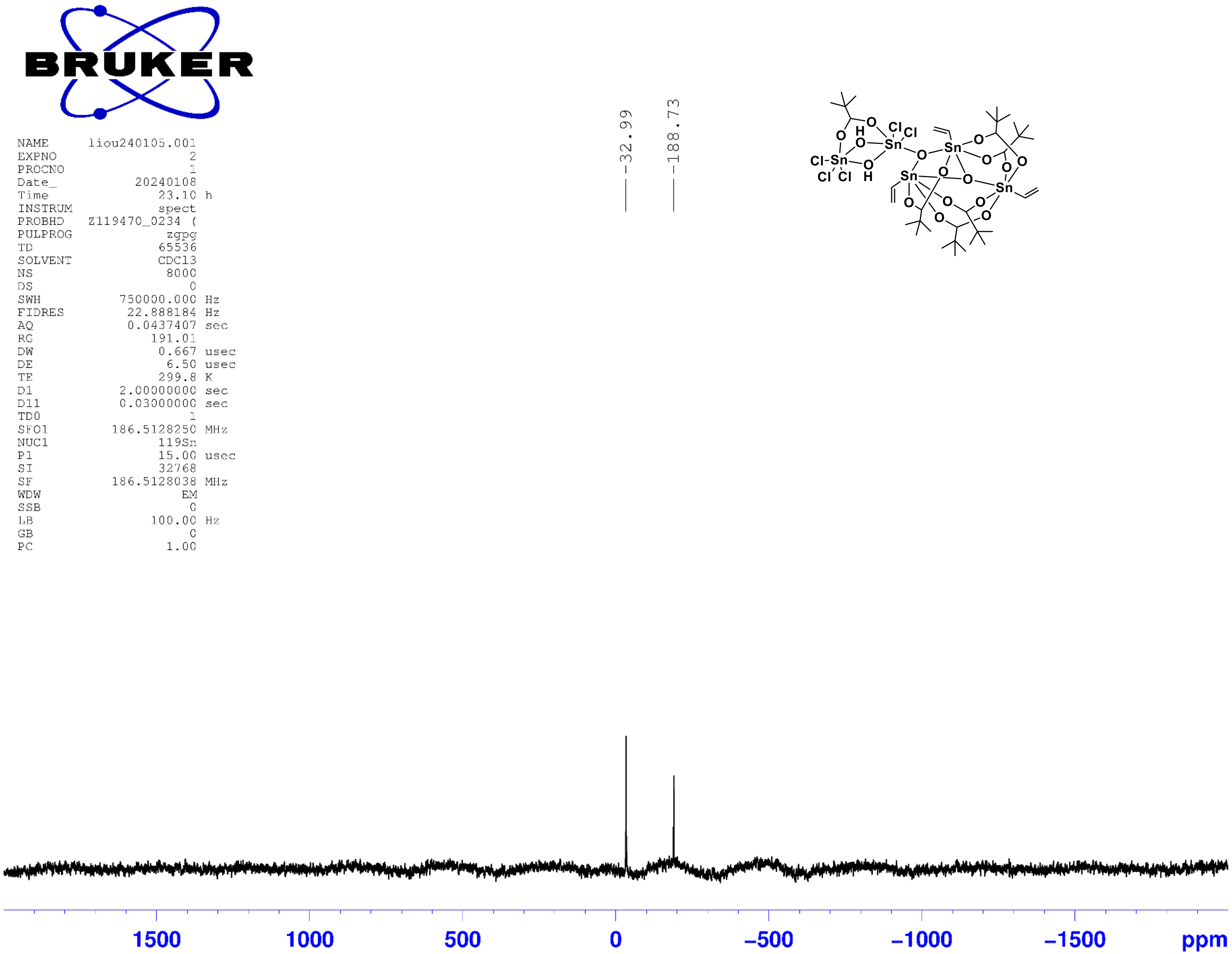

Figure S8. ^{119}Sn NMR of cluster 1

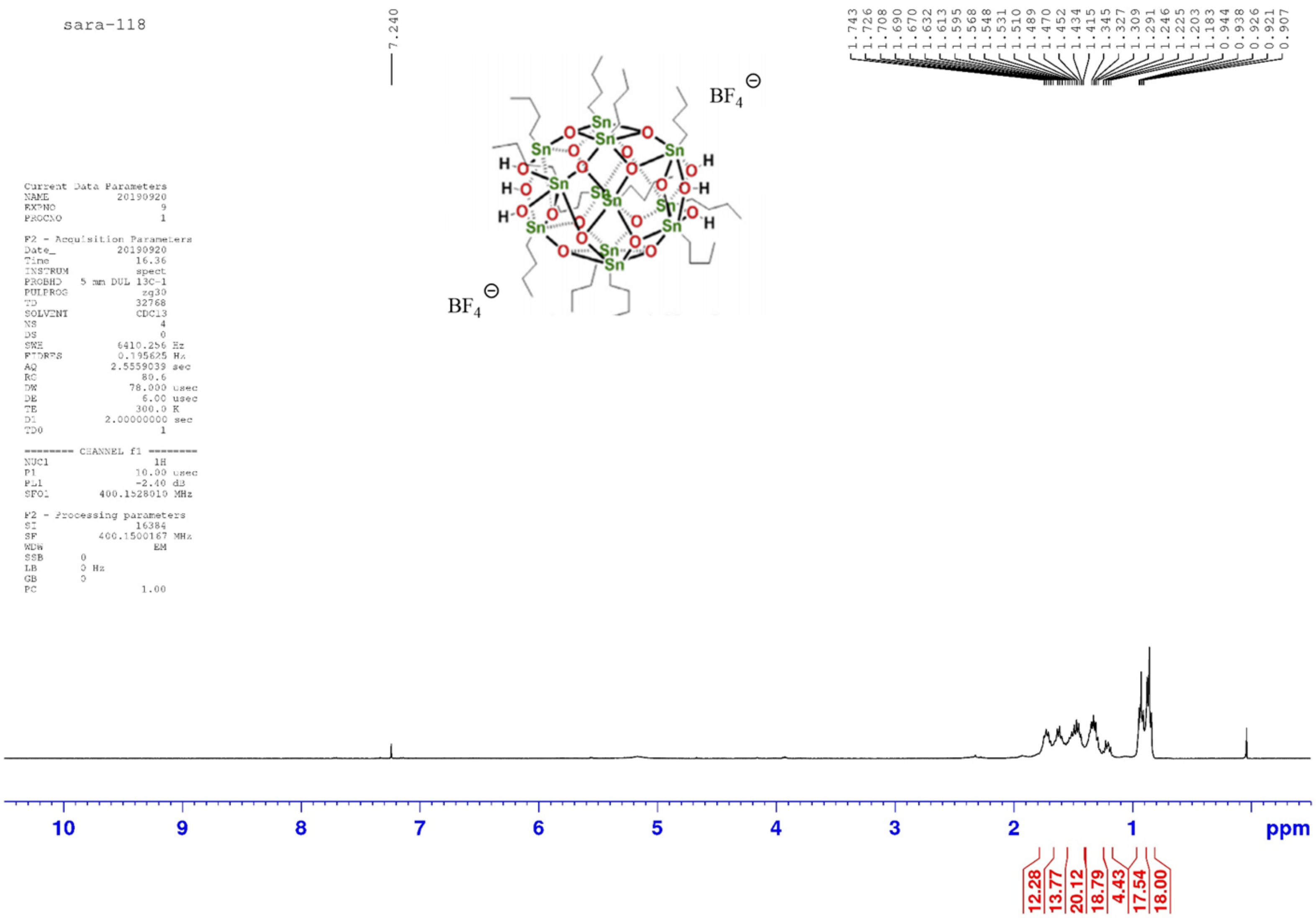

Figure S9. ^{1}H NMR of cluster 2

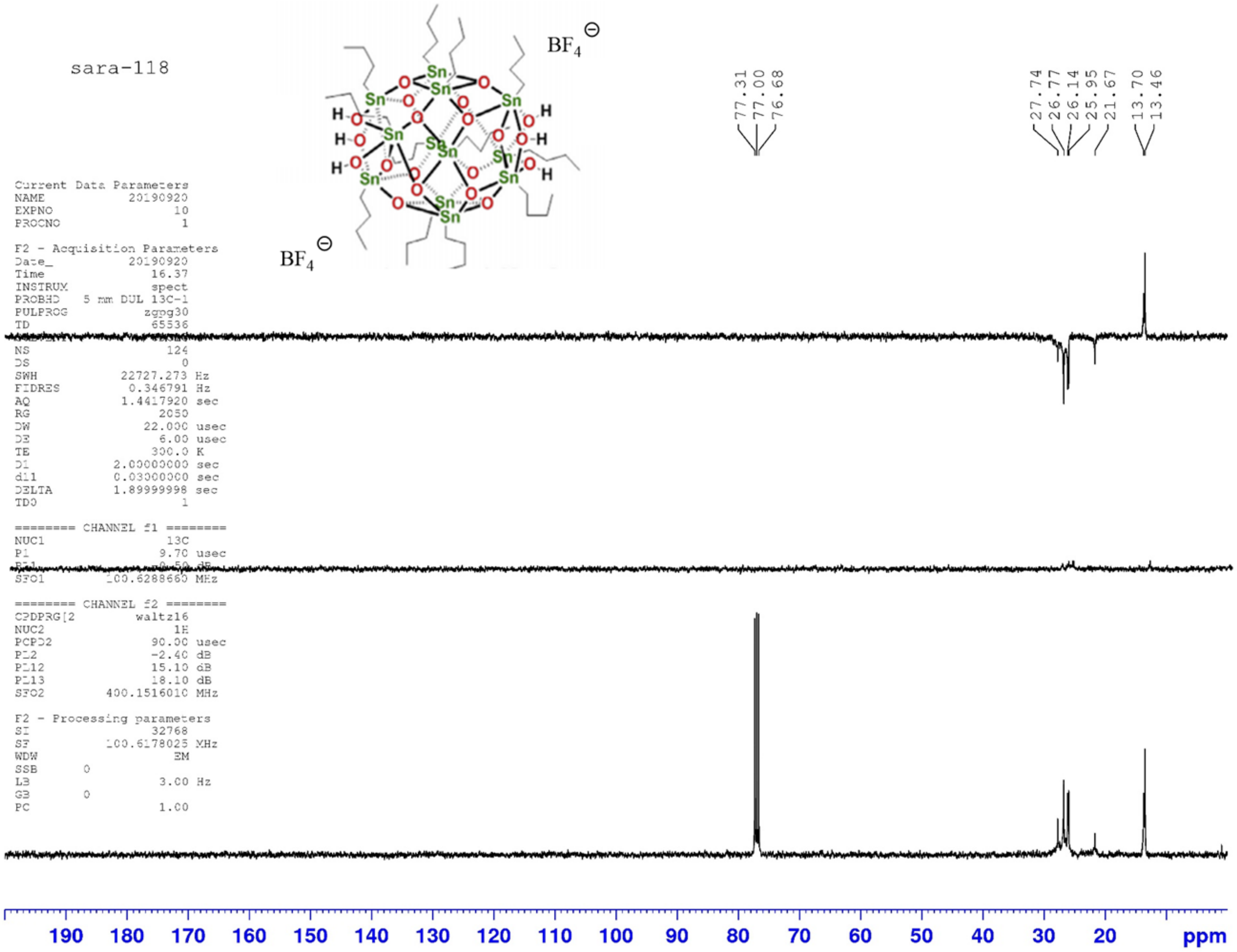

Figure S10. ^{13}C NMR of cluster 2

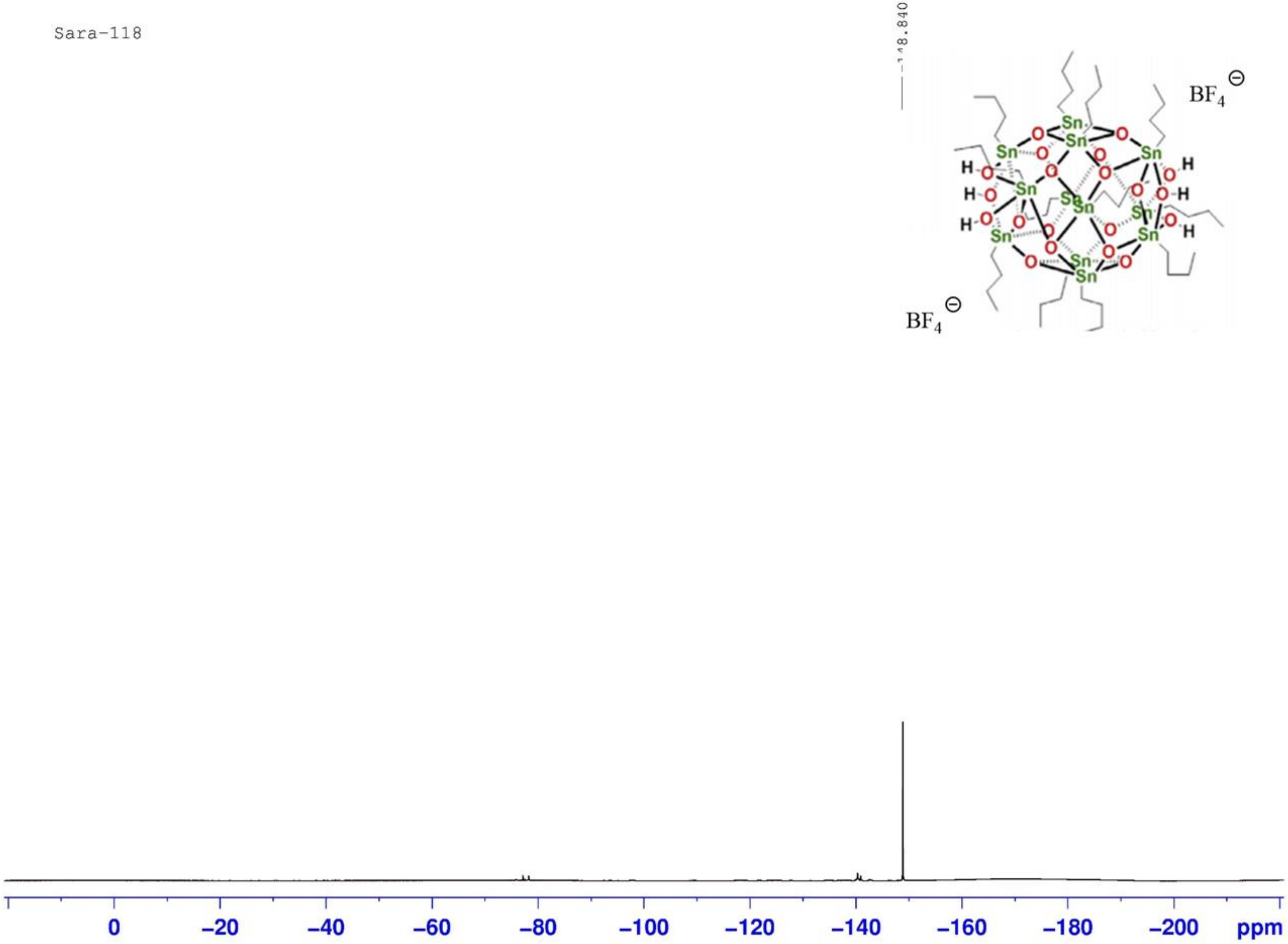

Figure S11. ^{19}F NMR of cluster 2

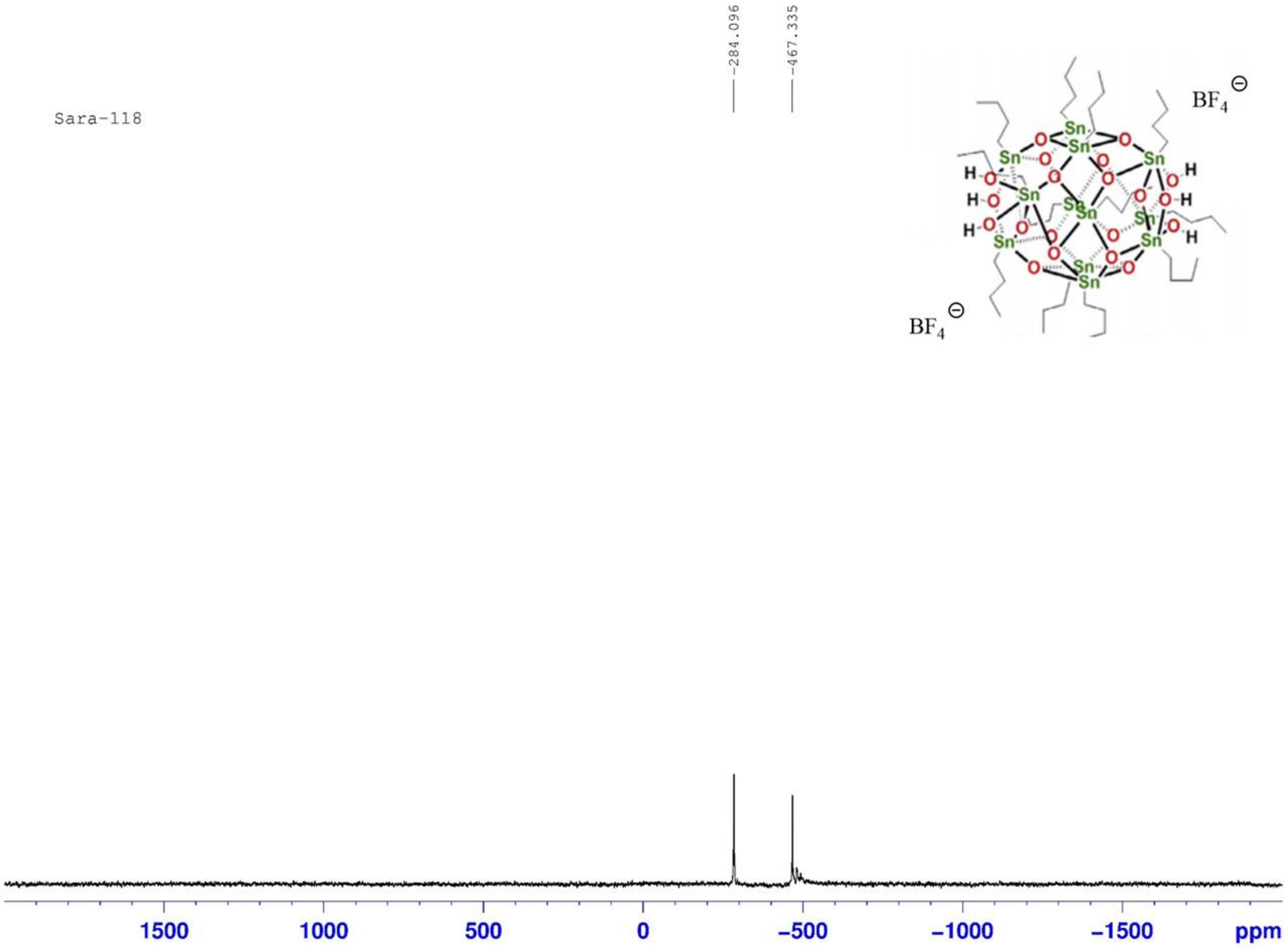

Figure S12. ^{119}Sn NMR of cluster 2
